# *Antrodia cinnamomea* Extract Attenuates Obesity by Targeting Adipogenic Pathways and Gut Dysbiosis in High-Fat Diet-Fed Mice

**DOI:** 10.3390/ijms26125856

**Published:** 2025-06-18

**Authors:** Kuen-Tze Lin, Shih-Yu Lee, Lee Ya-Jy, Po-Jui Wu, Tsu-Chung Chang, Wen-Liang Chang, I-Chuan Yen

**Affiliations:** 1 Department of Radiation Oncology, Cardinal Tien Hospital, New Taipei City 23148, Taiwan; 2 School of Medicine, College of Medicine, Fu-Jen Catholic University, New Taipei City 24205, Taiwan; 3 Graduate Institute of Aerospace and Undersea Medicine, National Defense Medical Center, Taipei 11490, Taiwan; 4 Department of Biochemistry, National Defense Medical Center, Taipei 11490, Taiwan; 5 School of Pharmacy, National Defense Medical Center, Taipei 11490, Taiwan

**Keywords:** *Antrodia cinnamomea*, adipogenesis, lipid metabolism, AMPK, gut microbiota, obesity

## Abstract

Obesity is a major metabolic disorder driven by excessive adipogenesis and lipid accumulation. This study investigated the anti-obesity effects and molecular mechanisms of *Antrodia cinnamomea* alcohol extract (ACE) in 3T3-L1 preadipocytes and a high-fat diet (HFD)-induced obesity mouse model. In vitro, *Antrodia cinnamomea* alcohol extract significantly inhibited adipocyte differentiation and lipid accumulation in 3T3-L1 cells by downregulating PPARγ and C/EBPα, while activating the AMPK pathway and suppressing MAPK signaling. In vivo, *Antrodia cinnamomea* alcohol extract administration reduced body weight, adipose tissue mass, and liver lipid accumulation in high-fat diet-fed mice, ameliorating non-alcoholic fatty liver disease (NAFLD) symptoms. Transcriptomic analysis of adipose tissue revealed that *Antrodia cinnamomea* alcohol extract modulated key gene expression profiles related to fatty acid metabolism and adipogenesis, suppressing lipid synthesis while enhancing β-oxidation. Furthermore, *Antrodia cinnamomea* alcohol extract rebalanced gut microbiota, increasing beneficial bacterial populations such as *Akkermansia* and *Bifidobacterium*, while reducing pro-inflammatory *Escherichia-Shigella* species. These findings demonstrate that *Antrodia cinnamomea* alcohol extract exerts multifaceted anti-obesity effects by regulating lipid metabolism, adipogenesis pathways, and gut microbiota composition, highlighting its potential as a natural therapeutic agent for obesity management.

## 1. Introduction

Obesity is a complex metabolic disorder characterized by excessive fat accumulation, which significantly increases the risk of developing cardiovascular diseases, type 2 diabetes, non-alcoholic fatty liver disease (NAFLD), and hypertension [1,2,3]. The pathogenesis of obesity is multifactorial, involving excessive caloric intake, sedentary lifestyles, genetic predisposition, and metabolic dysfunction [4]. Central to obesity-related metabolic disturbances is adipogenesis, the process by which preadipocytes differentiate into mature adipocytes, leading to excessive lipid accumulation in adipose tissue [5,6]. One of the key pathways involved in lipid metabolism and adipogenesis is the sterol regulatory element-binding protein (SREBP) pathway, which regulates the transcription of genes related to fatty acid and cholesterol synthesis. Specifically, SREBP-1 promotes fatty acid synthase (FASN) expression, contributing to triglyceride accumulation in adipocytes, while SREBP-2 primarily controls cholesterol homeostasis [7,8]. Dysregulation of these pathways exacerbates lipid accumulation and metabolic disturbances, reinforcing the need for effective therapeutic strategies.

The AMP-activated protein kinase (AMPK) pathway plays a central role in maintaining energy homeostasis and lipid metabolism. Activated AMPK inhibits adipogenesis by downregulating SREBP-1 and FASN expression, thereby reducing lipogenesis and promoting fatty acid oxidation [9]. Additionally, AMPK modulates key transcription factors such as peroxisome proliferator-activated receptor gamma (PPARγ) and CCAAT/enhancer-binding protein alpha (C/EBPα), both of which are essential regulators of adipocyte differentiation [10]. High AMPK activity has also been reported to enhance phosphorylation of Forkhead box protein O1 (FOXO1) [11,12], promoting its degradation and thereby relieving its inhibitory effects on PPARγ and C/EBPα [13], indirectly facilitating adipocyte differentiation. Moreover, activation of the mitogen-activated protein kinase (MAPK) signaling pathway―including extracellular signal-regulated kinase (ERK) and p38 MAPK (p38)―has also been shown to enhance adipogenesis by upregulating the expression of PPARγ and C/EBPα during the differentiation of 3T3-L1 preadipocytes [14,15]. In addition, JNK signaling also plays a role in adipogenesis: reduced JNK activity increases insulin sensitivity by decreasing IRS-1 Ser307 phosphorylation, whereas activated JNK1 can stabilize C/EBPα by inhibiting its ubiquitination, thereby enhancing its transactivation and DNA-binding capacity [15,16]. These transcription factors coordinate the transcriptional network that drives preadipocyte differentiation into mature adipocytes, making them critical targets in obesity management [17,18].

Natural products have gained increasing attention for their potential in managing obesity and related metabolic disorders. *Antrodia cinnamomea* (AC), a medicinal fungus native to Taiwan [19], has been traditionally used for its hepatoprotective, anti-inflammatory, and antioxidative properties [20,21,22]. Recent studies have demonstrated that AC can inhibit adipogenesis, suppress lipid accumulation, and improve metabolic function [23]. Specifically, AC extracts have been shown to downregulate PPARγ and C/EBPα, thereby preventing adipocyte differentiation. Moreover, AC influences lipid metabolism by activating AMPK and suppressing SREBP-1 activity, leading to reduced FASN expression and lower triglyceride synthesis [24]. Additionally, AC has been reported to modulate gut microbiota composition, further contributing to its metabolic benefits [25].

Despite accumulating evidence on the metabolic benefits of AC, its precise molecular mechanisms in obesity management remain incompletely understood. In this study, we investigated the impact of *Antrodia cinnamomea* alcohol extract on adipogenesis and lipid metabolism using 3T3-L1 preadipocytes and a high-fat diet (HFD)-induced obesity mouse model. We examined the effects of *Antrodia cinnamomea* alcohol extract on key metabolic regulators, including SREBP-1, FASN, AMPK, PPARγ, and C/EBPα, to elucidate its role in lipid metabolism and adipocyte differentiation. By identifying the molecular mechanisms through which *Antrodia cinnamomea* alcohol extract influences adipogenesis, this study aims to provide further insight into its potential as a natural therapeutic agent for obesity management.

## 2. Results

### 2.1. Antrodia cinnamomea Alcohol Extract Inhibits Adipogenesis and Lipid Accumulation in 3T3-L1 Cells

**Figure 1:**
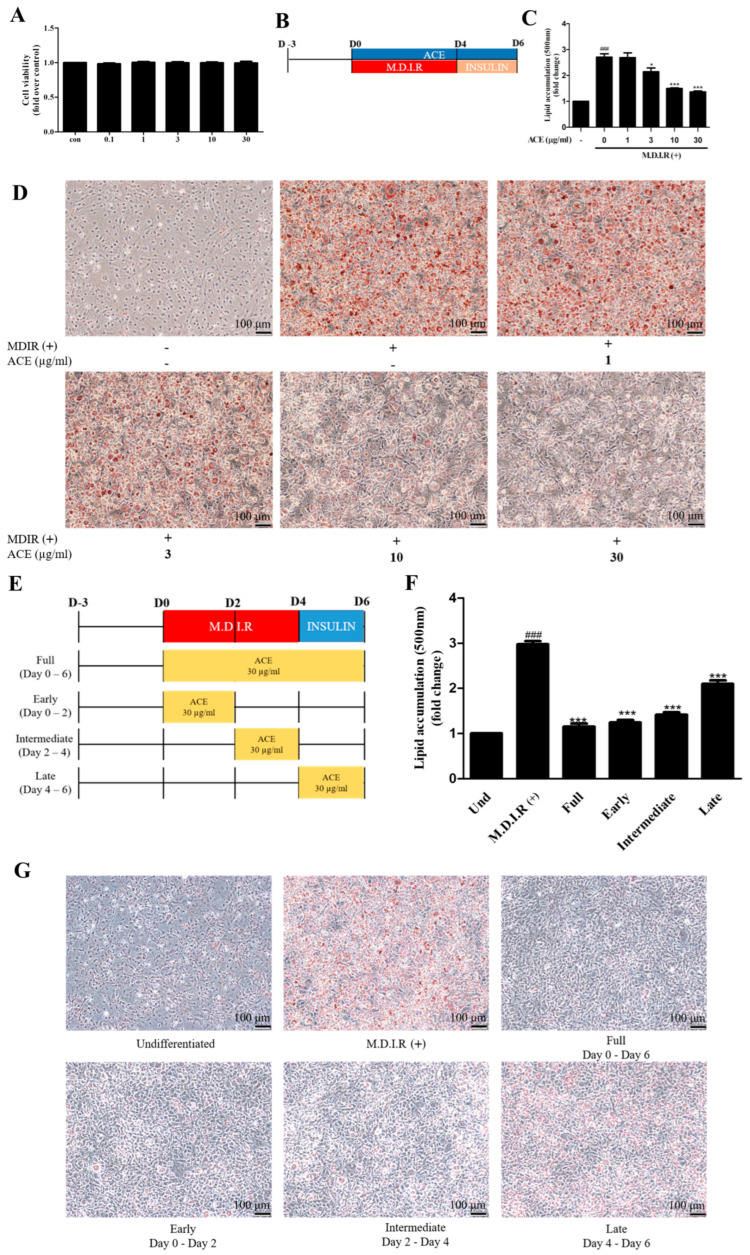
*Antrodia cinnamomea* alcohol extract reduces lipid accumulation in 3T3-L1 adipocytes. (**A**) Cell viability of 3T3-L1 preadipocytes treated with various concentrations of *Antrodia cinnamomea* alcohol extract for 24 h, assessed by CCK-8 assay. (**B**) Experimental timeline of 3T3-L1 adipocyte differentiation using M.D.I.R (3-isobutyl-1-methylxanthine, dexamethasone, insulin, rosiglitazone) for 4 days followed by insulin-only medium for 2 days. (**C**) Quantification of lipid content by extracting oil red O dye and measuring absorbance at 490 nm. (**D**) Oil red O staining was performed on day 6 to visualize intracellular lipid accumulation. (**E**) Schematic of experimental design for stage-specific *Antrodia cinnamomea* alcohol extract treatment during differentiation (early, intermediate, late, or full 6-day period). (**F**) Quantification of lipid accumulation under different *Antrodia cinnamomea* alcohol extract treatment windows. (**G**) Representative microscopy images showing lipid accumulation in each group. All images were captured at 200× magnification. Data are presented as mean ± SEM (n = 3). ### *p* < 0.001 vs. undifferentiated; * *p* < 0.05, *** *p* < 0.001 vs. M.D.I.R (+)-only control.

To determine the cytotoxicity of *Antrodia cinnamomea* alcohol extract in 3T3-L1 cells, cell viability assays were performed. Treatment with *Antrodia cinnamomea* alcohol extract (0.1–30 μg/mL) for 24 h did not affect 3T3-L1 cell viability (Figure 1A), indicating these concentrations are non-cytotoxic. To assess the anti-adipogenic effects of *Antrodia cinnamomea* alcohol extract, 3T3-L1 preadipocytes were differentiated using the standard six-day protocol (Figure 1B, M.D.I.R for 4 days followed by insulin for 2 days, hereafter referred to as M.D.I.R (+)) in the presence of various concentrations of *Antrodia cinnamomea* alcohol extract. Oil red O staining showed a dose-dependent reduction in intracellular lipid accumulation following *Antrodia cinnamomea* alcohol extract treatment (Figure 1C,D), suggesting its inhibitory effect on adipogenesis. To further elucidate the stage-specific effects of *Antrodia cinnamomea* alcohol extract, cells were treated with *Antrodia cinnamomea* alcohol extract (30 μg/mL) during distinct differentiation phases: early (day 0–2), intermediate (day 2–4), late (day 4–6), or the full 6-day period (Figure 1E). Compared to cells treated with differentiation medium alone (M.D.I.R), all *Antrodia cinnamomea* alcohol extract-treated groups showed significant reductions in lipid accumulation (Figure 1F,G). Notably, *Antrodia cinnamomea* alcohol extract treatment during the early stage (days 0–2) exhibited the most pronounced effect, closely approximating the efficacy of full-period *Antrodia cinnamomea* alcohol extract exposure. These findings suggest that *Antrodia cinnamomea* alcohol extract exerts inhibitory effects throughout adipogenesis, with the early phase being the most responsive period.

### 2.2. Antrodia cinnamomea Alcohol Extract Modulates Adipogenic Signaling Pathways in 3T3-L1 Cells

**Figure 2:**
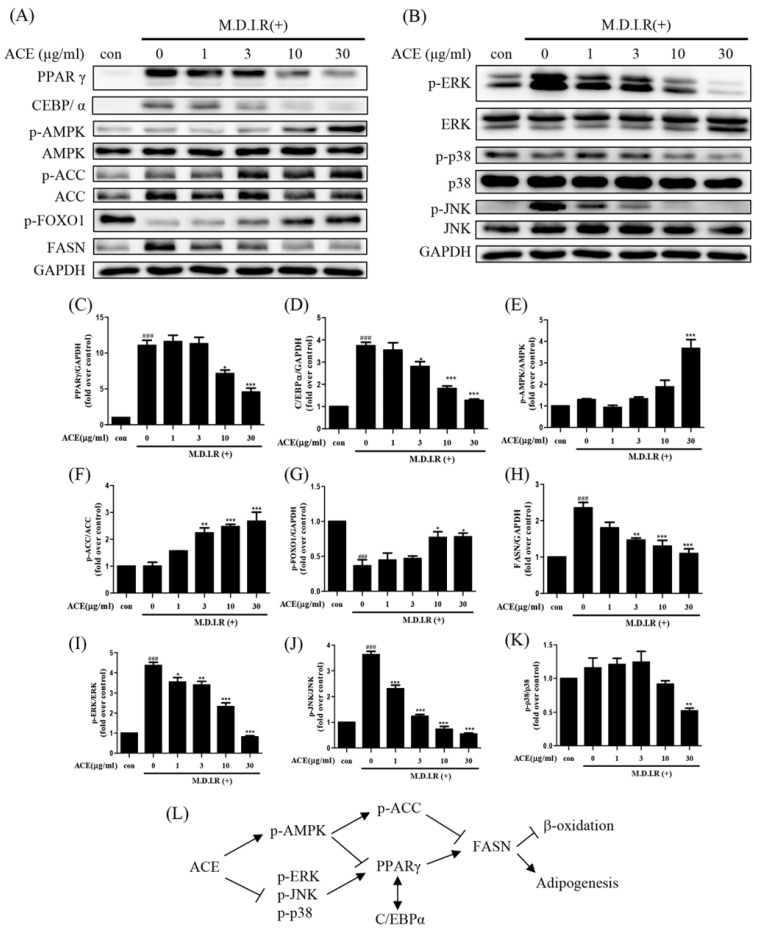
*Antrodia cinnamomea* alcohol extract modulates adipogenic and metabolic regulators in 3T3-L1 adipocytes. (**A**,**B**) Representative Western blot images of indicated protein expression at day 2 of differentiation. (**C**–**K**) Quantification of PPARγ, C/EBPα, p-AMPK, p-ACC, p-FOXO1, FASN, p-ERK, p-JNK, and p-p38 protein levels, respectively. (**L**) Diagram summarizing the regulatory effects of *Antrodia cinnamomea* alcohol extract on adipogenic and metabolic signaling pathways. GAPDH was used as an internal control. Quantification of protein levels was normalized to GAPDH, and phospho-protein levels―except p-FOXO―were normalized to their respective total proteins. Data are from three independent experiments. ### *p* < 0.001 vs. undifferentiated; * *p* < 0.05, ** *p* < 0.01,*** *p* < 0.001 vs. M.D.I.R (+).

To investigate the underlying mechanisms of *Antrodia cinnamomea* alcohol extract-mediated adipogenesis inhibition, we analyzed the expression of key transcription factors and metabolic regulators at day 2 of differentiation (early stage). Western blot analysis showed that *Antrodia cinnamomea* alcohol extract reduced the expression of key adipogenic transcription factors PPARγ and C/EBPα in a dose-dependent manner, both of which are critical for adipocyte maturation (Figure 2A,C,D). FASN, a downstream effector involved in lipid biosynthesis, was similarly decreased (Figure 2A,H). Concomitantly, *Antrodia cinnamomea* alcohol extract treatment significantly increased the phosphorylation levels of AMPK and its downstream target ACC (Figure 2A,E,F), indicative of metabolic reprogramming. Meanwhile, phosphorylated FOXO1 was also elevated (Figure 2A,G). In terms of MAPK signaling, *Antrodia cinnamomea* alcohol extract significantly reduced the phosphorylation of ERK and JNK in a dose-dependent manner (Figure 2B,I,J) while its effect on p38 phosphorylation was minimal (Figure 2B,K). These findings suggest that *Antrodia cinnamomea* alcohol extract activates the AMPK signaling pathway while inhibiting ERK activity during early adipogenesis. Together, these results support the notion that *Antrodia cinnamomea* alcohol extract suppresses adipocyte differentiation by modulating both transcriptional and signaling mechanisms, primarily through early-stage intervention (Figure 2L).

### 2.3. Antrodia cinnamomea Alcohol Extract Reduces High-Fat Diet-Induced Body Weight Gain and Improves Serum Biochemical Parameters in Mice

**Figure 3:**
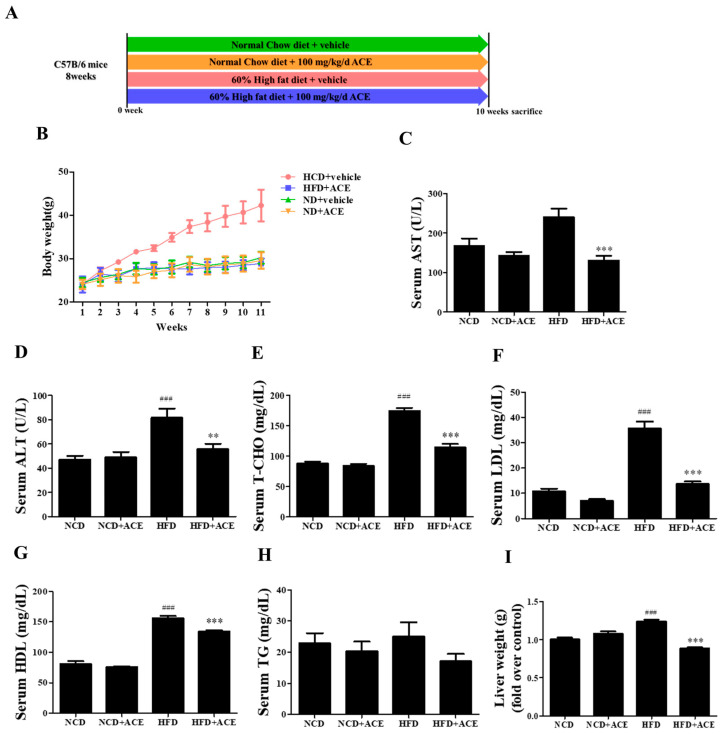
*Antrodia cinnamomea* alcohol extract attenuates high-fat diet-induced weight gain and improves serum biochemical profiles. (**A**) Schematic of the animal study design with four groups: NCD, NCD + ACE, HFD, and HFD + ACE. (**B**) Body weight recorded weekly for 10 weeks. (**C**,**D**) Serum AST and ALT levels measured at study endpoint. (**E**–**H**) Serum lipid profiles including total cholesterol (T-CHO), LDL, HDL, and triglycerides (TG). (**I**) Liver weight measured post-sacrifice. Data are shown as mean ± SEM. ### *p* < 0.001 vs. NCD; ** *p* < 0.01, *** *p* < 0.001 vs. high-fat diet.

To examine the anti-obesity potential of *Antrodia cinnamomea* alcohol extract in vivo, C57BL/6 mice were divided into four groups and treated for 10 weeks: normal chow diet (NCD), normal chow diet with *Antrodia cinnamomea* alcohol extract (NCD + ACE), high-fat diet (HFD), and high-fat diet with *Antrodia cinnamomea* alcohol extract (HFD + ACE) (Figure 3A). Mice fed an high-fat diet displayed significant body weight gain compared to the normal chow diet group, whereas *Antrodia cinnamomea* alcohol extract supplementation markedly attenuated this increase (Figure 3B).

Serum biochemical analysis revealed that high-fat diet feeding elevated AST and ALT levels, indicative of liver stress or damage. These elevations were significantly reversed by *Antrodia cinnamomea* alcohol extract treatment (Figure 3C,D). high-fat diet also led to increased levels of total cholesterol (T-CHO), LDL, and HDL (Figure 3E–G), which were significantly reduced in the HFD + ACE group. Although triglyceride (TG) levels were modestly reduced in HFD + ACE mice, the change did not reach statistical significance (Figure 3H). Additionally, liver weights were increased by high-fat diet feeding, and this effect was ameliorated with *Antrodia cinnamomea* alcohol extract supplementation (Figure 3I).

### 2.4. Antrodia cinnamomea Alcohol Extract Attenuates Hepatic Steatosis and Non-Alcoholic Fatty Liver Disease-like Features Induced by High-Fat Diet

**Figure 4:**
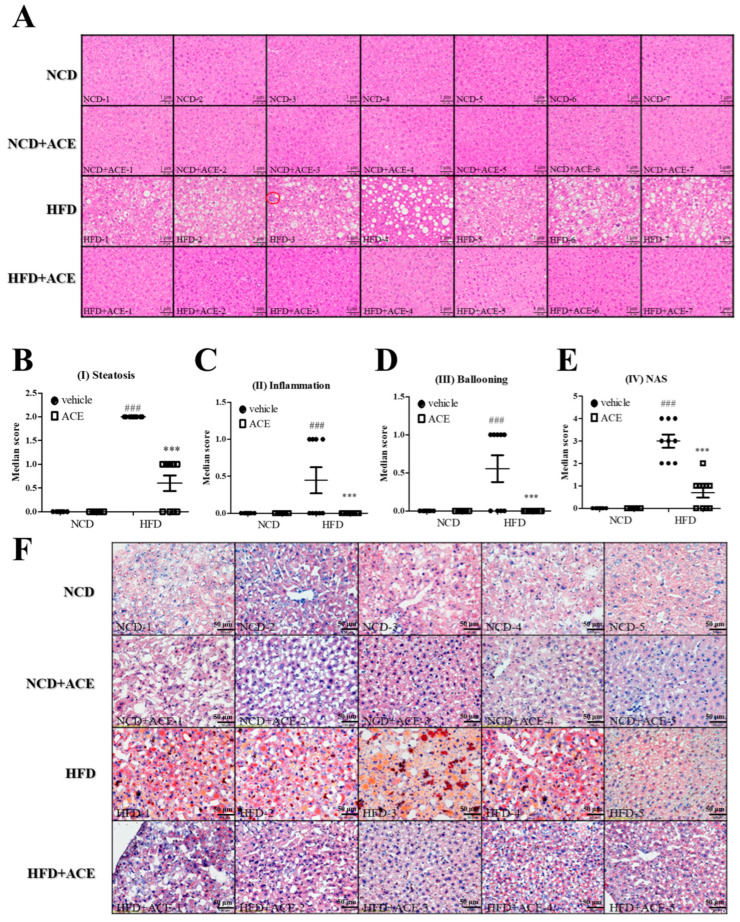
*Antrodia cinnamomea* alcohol extract improves hepatic histology and reduces non-alcoholic fatty liver disease-related pathology. (**A**) Representative H&E-stained liver sections from each group. (**B**–**D**) Histological scoring of steatosis, immune cell infiltration (inflammation), and hepatocellular ballooning using established non-alcoholic fatty liver disease criteria. (**E**) Composite non-alcoholic fatty liver disease Activity Score (NAS) calculated from the above features. (**F**) Oil red O staining of liver sections to assess lipid accumulation. Corresponding histological features are schematically illustrated in Figure S4. Data are shown as mean ± SEM (n = 5–10). ### *p* < 0.001 vs. NCD; *** *p* < 0.001 vs. high-fat diet.

Histological examination of liver sections stained with hematoxylin and eosin (H&E) revealed that mice fed an high-fat diet developed classic features of non-alcoholic fatty liver disease, including macrovesicular steatosis, immune cell infiltration, and hepatocellular ballooning (Figure 4A; corresponding histological features are schematically illustrated in Figure S4). These features were substantially alleviated in the HFD + ACE group. Quantitative scoring of individual non-alcoholic fatty liver disease features showed that steatosis (Figure 4B), inflammation (Figure 4C), and ballooning (Figure 4D) were all significantly elevated in high-fat diet-fed mice, and were significantly improved in mice receiving *Antrodia cinnamomea* alcohol extract. The composite NAS score, which sums the scores of steatosis, inflammation, and ballooning to assess overall disease severity [26], was also significantly reduced in HFD + ACE mice (Figure 4E). Consistent with the histology, oil red O staining demonstrated prominent lipid accumulation in hepatocytes from high-fat diet-fed mice, which was visibly reduced by *Antrodia cinnamomea* alcohol extract treatment (Figure 4F).

### 2.5. Antrodia cinnamomea Alcohol Extract Reduces Adipose Tissue Accumulation and Adipocyte Hypertrophy in High-Fat Diet-Fed Mice

**Figure 5:**
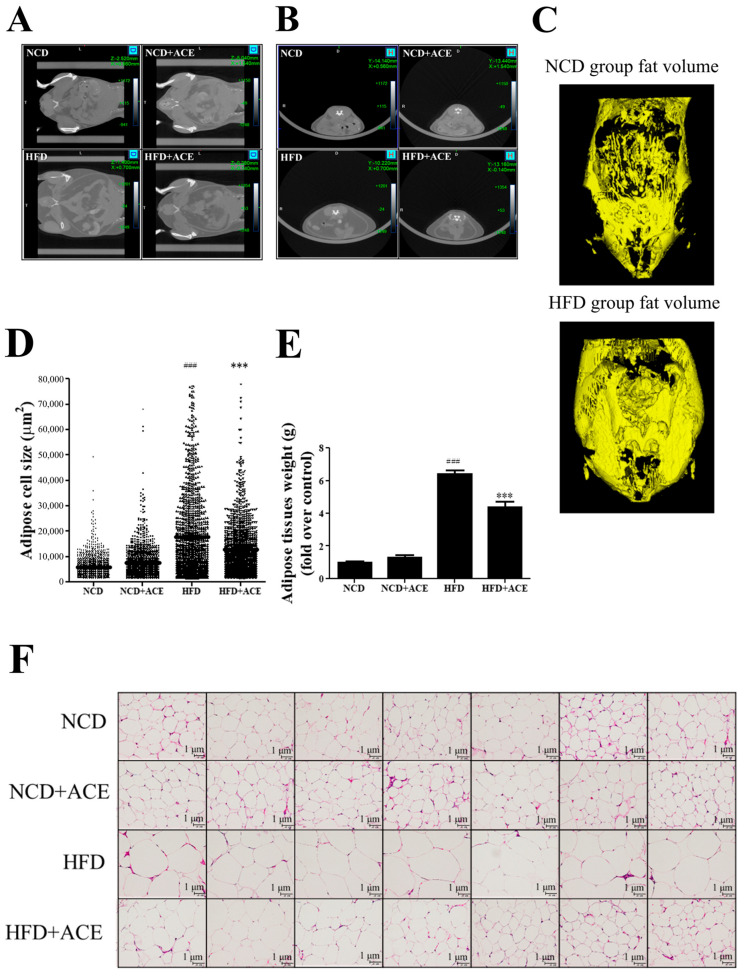
*Antrodia cinnamomea* alcohol extract reduces visceral fat accumulation and adipocyte hypertrophy in high-fat diet-fed mice. Transverse (**A**) and coronal (**B**) micro-CT images of abdominal regions from each group. (**C**) Three-dimensional reconstructed visualization of abdominal fat volume. (**D**) Quantification of adipocyte size from H&E-stained paratesticular adipose tissue. (**E**) Weight of dissected paratesticular adipose tissue. (**F**) Representative histological images of H&E-stained paratesticular adipose tissue showing adipocyte morphology. Data are shown as mean ± SEM. ### *p* < 0.001 vs. NCD; *** *p* < 0.001 vs. high-fat diet.

To assess the impact of *Antrodia cinnamomea* alcohol extract on fat deposition, micro-computed tomography (micro-CT) was performed to visualize abdominal fat distribution in mice from each group. Transverse (Figure 5A) and coronal (Figure 5B) views showed pronounced abdominal fat accumulation in high-fat diet-fed mice, which appeared substantially reduced in the HFD + ACE group. Additionally, three-dimensional reconstruction of abdominal fat tissue illustrated visually larger fat volumes in high-fat diet-fed mice, while *Antrodia cinnamomea* alcohol extract supplementation appeared to reduce overall fat expansion (Figure 5C). Moreover, histological analysis of the paratesticular adipose tissue revealed significant adipocyte hypertrophy in high-fat diet mice, which was markedly ameliorated by *Antrodia cinnamomea* alcohol extract treatment (Figure 5F). Quantification confirmed that *Antrodia cinnamomea* alcohol extract significantly reduced both mean adipocyte diameter (Figure 5D) and tissue weight (Figure 5E) compared to the high-fat diet group.

### 2.6. Antrodia cinnamomea Alcohol Extract Regulates Adipogenic and Metabolic Protein Expression in Adipose Tissue

**Figure 6:**
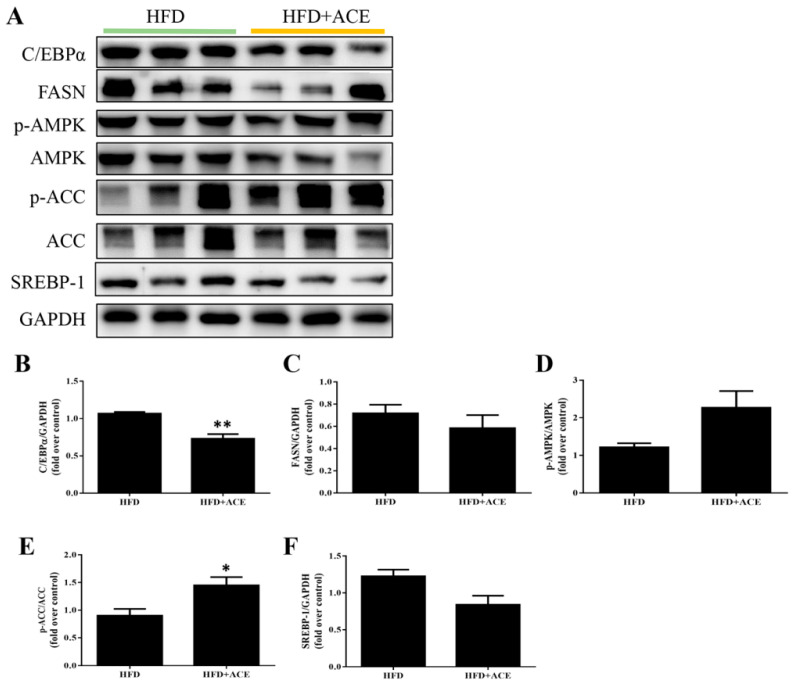
*Antrodia cinnamomea* alcohol extract regulates adipogenic and metabolic protein expression in paratesticular adipose tissue. (**A**) Representative Western blot images from paratesticular adipose tissue of HFD and HFD + ACE mice. (**B**–**F**) Quantification of C/EBPα, FASN, p-AMPK, p-ACC, and SREBP-1 protein levels. GAPDH was used as the internal control. Quantification of the indicated protein expression was normalized to GAPDH, and phospho-protein levels were normalized to their respective unphosphorylated proteins. Data are shown as mean ± SEM from three independent experiments. * *p* < 0.05, ** *p* < 0.01 vs. high-fat diet.

To evaluate the effect of *Antrodia cinnamomea* alcohol extract on fat metabolism in vivo, protein expression in paratesticular adipose tissue was analyzed by Western blotting (Figure 6A). Compared to the high-fat diet group, *Antrodia cinnamomea* alcohol extract treatment significantly reduced the levels of C/EBPα and FASN, which are key regulators of adipocyte differentiation and lipid synthesis, respectively (Figure 6B,C). Additionally, *Antrodia cinnamomea* alcohol extract treatment markedly increased the phosphorylation levels of AMPK and its downstream effector ACC (Figure 6D,E), indicating activation of the AMPK pathway in adipose tissue of *Antrodia cinnamomea* alcohol extract-treated mice. Moreover, SREBP-1, another transcriptional regulator involved in lipogenesis, showed a decreasing trend in the HFD + ACE group (Figure 6F). Although not all changes reached statistical significance, the overall trends were consistent with those observed in the in vitro results. These findings suggest that *Antrodia cinnamomea* alcohol extract may reduce fat accumulation in adipose tissue by modulating the expression of key metabolic regulators.

### 2.7. Transcriptomic Profiling of Paratesticular Adipose Tissue Following Antrodia cinnamomea Alcohol Extract Treatment

**Figure 7:**
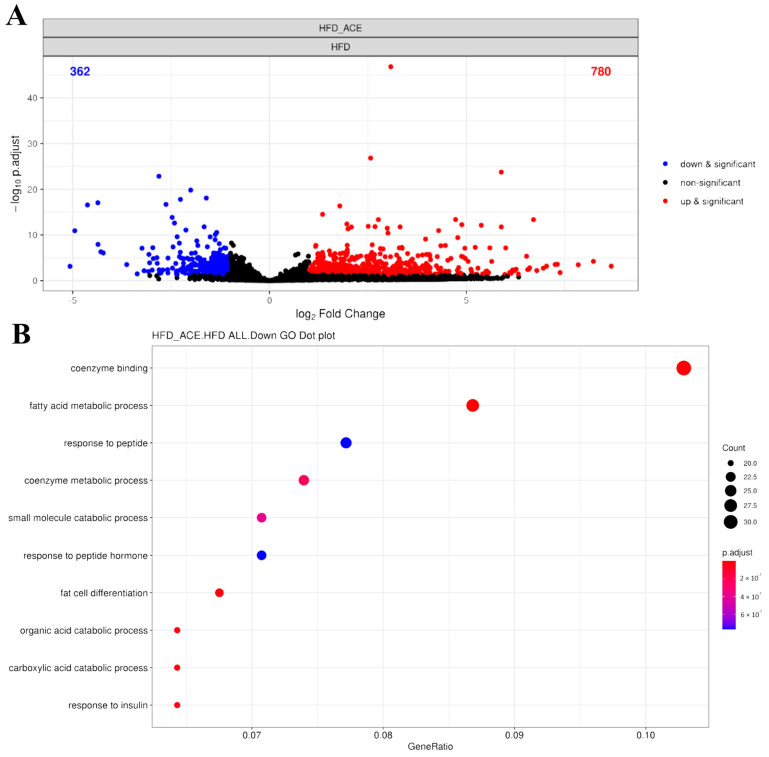
RNA sequencing analysis of paratesticular adipose tissue from *Antrodia cinnamomea* alcohol extract-treated high-fat diet mice. (**A**) Volcano plot displaying differentially expressed genes (DEGs) between HFD and HFD + ACE groups. Red and blue dots indicate significantly upregulated and downregulated genes in HFD + ACE mice, respectively (|log_2_ FC| ≥ 1, adjusted *p* < 0.05). (**B**) Gene ontology (GO) enrichment analysis of significantly downregulated DEGs. Top 10 enriched GO biological process terms are shown, primarily related to fatty acid metabolism and adipocyte differentiation.

To explore the gene expression changes associated with *Antrodia cinnamomea* alcohol extract treatment in obese mice, transcriptome analysis was performed using paratesticular adipose tissue from HFD and HFD + ACE groups. A total of 16,114 genes were detected by RNA sequencing. Principal component analysis (PCA) revealed a clear separation between the HFD and HFD + ACE groups, indicating distinct gene expression profiles between the two conditions (Figure S1A). Differential expression analysis identified 780 genes upregulated and 362 genes downregulated in the HFD + ACE group compared to the HFD group, as visualized in the volcano plot (Figure 7A). Heatmap visualization also highlighted consistent expression clustering within groups (Figure S1B).

Gene ontology analysis showed that *Antrodia cinnamomea* alcohol extract treatment suppressed multiple biological processes related to fatty acid metabolic process and fat cell differentiation (Figure 7B, Tables S1 and S2). Representative genes associated with fatty acid metabolism included *Acsm3*, *Cyp2e1*, *Pck1*, *Gpam*, *Acadsb*, and *Acaca*. Downregulated genes related to adipocyte differentiation included *Cebpd*, *Scd1*, *Resistin*, and *Adig* (adipogenin). The top 10 significantly upregulated and downregulated genes affected by *Antrodia cinnamomea* alcohol extract were identified (Tables S3 and S4). Among them, several genes, such as *Cck*, *Gpr50*, *Grem2*, *Fam13a*, *Irf4*, and *Acsm3*, are associated with metabolism-related processes, suggesting that *Antrodia cinnamomea* alcohol extract modulates fat metabolic pathways at the transcriptional level.

### 2.8. Antrodia cinnamomea Alcohol Extract Modulates the Gut Microbiota in High-Fat Diet-Fed Mice

To investigate the impact of *Antrodia cinnamomea* alcohol extract on gut microbiota composition, 16S rRNA sequencing was performed in fecal samples from four experimental groups. Figure S2A shows a heatmap of pairwise dissimilarity in gut microbiota composition among all samples. Principal component analysis (PCA) demonstrated distinct clustering of gut microbiota compositions among the four groups (Figure S2B), with *Antrodia cinnamomea* alcohol extract treatment leading to notable shifts in both normal chow diet and high-fat diet contexts.

**Figure 8:**
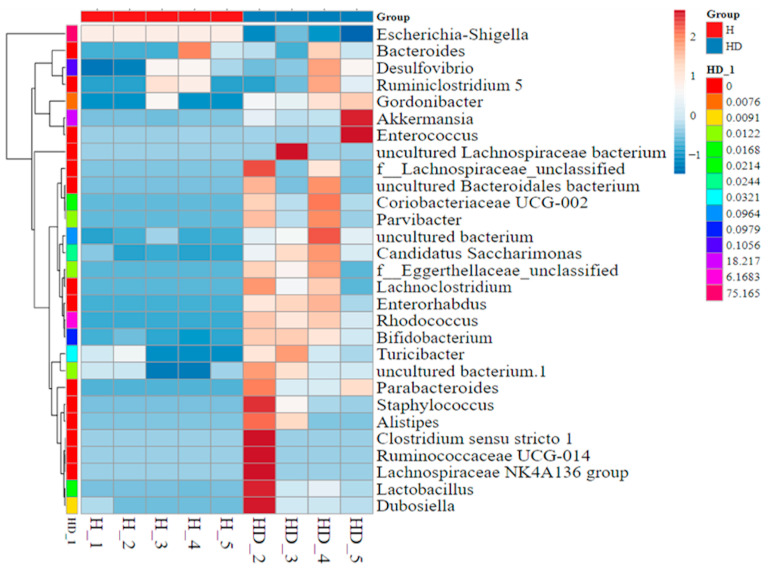
Heatmap of differentially abundant gut microbiota identified by LEfSe analysis in high-fat diet-fed mice treated with *Antrodia cinnamomea* alcohol extract. Linear discriminant analysis (LDA) effect size (LEfSe) analysis was performed to identify bacterial taxa that differed significantly between the HFD and HFD + ACE groups. This heatmap displays the relative abundance of selected discriminative taxa across individual mice in each group. Each column represents a sample; each row represents a taxon at the genus or higher taxonomic level. Color intensity indicates relative abundance (see scale bar), with red denoting higher abundance and blue denoting lower abundance. Hierarchical clustering of samples (left dendrogram) illustrates similarities in gut microbiota composition across treatments. Group identity of each sample is indicated by the color key along the top axis. H indicates the HFD group; HD indicates the HFD + ACE group. The numbers following each group label represent individual sample numbers.

In high-fat diet-fed mice, *Antrodia cinnamomea* alcohol extract increased the relative abundance of 12 bacterial taxa, including *Akkermansia*, *Bifidobacterium*, *Lactococcus*, *Muribaculaceae*, *Gordonibacter*, and *Turicibacter* (Figure 8, Table S5). Previous studies have reported reduced levels of *Akkermansia*, *Bifidobacterium*, and *Lactobacillus* in high-fat diet-fed mice compared to standard diet controls [27,28,29]. Conversely, the abundance of *Escherichia–Shigella*, a genus frequently associated with intestinal inflammation and metabolic disorders [27,30], was notably reduced following *Antrodia cinnamomea* alcohol extract treatment. Linear discriminant analysis effect size analysis further delineated the relative abundance of different microbiota phyla in each group (Figure 8 and Figure S2C). These findings suggest a shift in gut microbiota composition toward a more beneficial and anti-inflammatory profile in *Antrodia cinnamomea* alcohol extract-treated mice.

Furthermore, Table S6 highlights gut microbial changes in the normal chow (NCD) diet group with or without *Antrodia cinnamomea* alcohol extract. Notably, several taxa increased in the NCD + ACE group, such as *Bacteroides*, *Enterorhabdus*, *Muribaculaceae*, and *Parabacteroides*, are also considered beneficial or anti-inflammatory in prior studies [31,32,33,34]. However, not all reductions should be interpreted as favorable. For instance, *Rhodococcus*, which was decreased in NCD + ACE but increased in HFD + ACE groups, is capable of degrading harmful sterols such as 7-ketocholesterol and may exert protective effects [35]. This indicates that the effects of *Antrodia cinnamomea* alcohol extract on microbial populations may be condition-dependent, with certain taxa showing opposite trends under different dietary backgrounds. The full list of significant changes is provided in Tables S5 and S6 and further group-wise distinctions are illustrated in Figure S3.

## 3. Discussion

In this study, we demonstrated that *Antrodia cinnamomea* alcohol extract (ACE) exerts potent anti-obesity effects in both 3T3-L1 preadipocyte and high-fat diet (HFD)-induced obese mouse models. Mechanistically, *Antrodia cinnamomea* alcohol extract inhibited adipogenesis by downregulating the expression of PPARγ and C/EBPα, suppressing MAPK activation, and enhancing AMPK phosphorylation. These molecular changes translated into reduced lipid accumulation, decreased adipocyte hypertrophy, and ameliorated liver steatosis. Moreover, transcriptomic and microbiome analyses uncovered broad modulation of lipid metabolic pathways and gut microbial composition, respectively, providing new insight into the multifaceted actions of *Antrodia cinnamomea* alcohol extract against obesity.

Although previous studies have demonstrated that *Antrodia cinnamomea* alcohol extract inhibits adipogenesis via PPARγ and C/EBPα modulation in 3T3-L1 cells [23], our study provides several novel contributions. First, we extended mechanistic investigations beyond the cellular level by performing Western blot and transcriptomic analysis directly in the paratesticular adipose tissue of high-fat diet-fed mice. This approach revealed in vivo changes in key adipogenic proteins (C/EBPα, FASN, SREBP-1) and transcript-level regulation of multiple lipid metabolic pathways, providing physiologically relevant evidence of *Antrodia cinnamomea* alcohol extract activity at the tissue level—an aspect that has been underexplored in prior *Antrodia cinnamomea* alcohol extract-related studies. Second, our study is among the few to comprehensively examine how *Antrodia cinnamomea* alcohol extract modulates gut microbiota composition using LEfSe and PCA analysis. While Chang et al. (2018) previously reported that *Antrodia cinnamomea* alcohol extract can reshape gut microbial communities [25], we provided deeper insight by showing taxonomic-level shifts (e.g., increases in *Akkermansia*, *Turicibacter*, and *Bifidobacterium*) and diet-dependent effects, as evidenced by the differential responses in both high-fat diet and NCD backgrounds. Lastly, the integrative experimental design of this study—combining in vitro differentiation assays, in vivo physiological and histological analysis, transcriptomic profiling, and microbiota sequencing—offers a comprehensive systems-level view of *Antrodia cinnamomea* alcohol extract’s anti-obesity mechanisms, enhancing both mechanistic clarity and translational relevance. While only male mice were used in this study to minimize potential hormonal effects on obesity-related parameters, we acknowledge that gender differences may influence adipogenesis and metabolic responses. This is a limitation in the current study, and future studies will include female mice to address potential sex-dependent effects of *Antrodia cinnamomea* alcohol extract treatment.

AMPK plays a pivotal role in metabolic regulation by inhibiting lipogenesis and promoting β-oxidation [10]. In our study, *Antrodia cinnamomea* alcohol extract significantly activated the AMPK/ACC axis in both 3T3-L1 cells and adipose tissue, consistent with prior reports that *Antrodia cinnamomea* modulates energy-sensing pathways to suppress lipid synthesis [24]. This activation likely contributes to the observed inhibition of adipogenesis and enhanced lipid metabolism.

In addition to AMPK, our data revealed that *Antrodia cinnamomea* alcohol extract also suppresses MAPK signaling. Western blot analysis showed that *Antrodia cinnamomea* alcohol extract significantly reduced the phosphorylation of ERK and JNK in a dose-dependent manner, whereas its effect on p38 phosphorylation was relatively minimal. Since both ERK and JNK are known to enhance adipogenesis by stabilizing and activating PPARγ and C/EBPα [14,15,16], their suppression suggests that *Antrodia cinnamomea* alcohol extract effectively interferes with key MAPK-driven adipogenic pathways. The relatively modest inhibition of p38 may reflect its broader involvement in cellular stress responses and its context-dependent role in adipogenesis—being promotive in early differentiation but dispensable or even inhibitory in later stages [36,37,38]. Rather than uniformly suppressing all MAPK branches, *Antrodia cinnamomea* alcohol extract appears to selectively target the pro-adipogenic arms, while leaving p38 activity relatively intact, which may help preserve cellular mechanisms involved in inflammation resolution and stress adaptation [39].

Together, these findings indicate that *Antrodia cinnamomea* alcohol extract regulates adipogenesis through dual modulation of AMPK activation and MAPK inhibition, highlighting its potential as a multi-targeted metabolic regulator.

Our RNA-seq analysis revealed that *Antrodia cinnamomea* alcohol extract treatment led to the downregulation of multiple key genes involved in fatty acid metabolism and adipocyte differentiation. Among the top downregulated pathways were those related to lipid synthesis and adipocyte maturation. Notably, *Scd1*, *Acaca*, *Pck1*, and *Adig* (adipogenin) were significantly suppressed in the adipose tissue of *Antrodia cinnamomea* alcohol extract-treated high-fat diet-fed mice. These genes are strongly implicated in lipid storage and triglyceride biosynthesis. For instance, *Adig* encodes a membrane-associated protein that is upregulated during adipogenesis and highly expressed in mature adipocytes [40]. Its expression was nearly threefold higher in high-fat diet mice compared to NCD mice, and *Antrodia cinnamomea* alcohol extract treatment effectively reduced its levels. This is consistent with previous findings showing that *Adig* knockout mice exhibit reduced fat accumulation and diminished PPARγ activity [41]. Similarly, suppression of *Scd1*, which catalyzes the formation of monounsaturated fatty acids and promotes triglyceride (TG) synthesis, has been associated with improved insulin sensitivity and reduced adipose inflammation [42,43]. Downregulation of *Cyp2e1*—a gene whose deficiency in mice improves glucose tolerance and energy expenditure—also supports the beneficial metabolic shift observed in *Antrodia cinnamomea* alcohol extract-treated mice [44]. Additionally, the suppression of *Pck1*, which participates in glyceroneogenesis and triglyceride synthesis; *Gpam*, which catalyzes the initial step in glycerolipid biosynthesis; and *Acaca*, a key enzyme for malonyl-CoA production, further indicates that *Antrodia cinnamomea* alcohol extract reprograms lipid metabolic pathways at the transcriptional level to reduce lipogenesis [45,46,47]. These findings suggest that *Antrodia cinnamomea* alcohol extract limits adipose tissue expansion in high-fat diet-induced obesity by targeting multiple nodes of the lipid biosynthetic network.

We also observed that *Antrodia cinnamomea* alcohol extract-treated high-fat diet-fed mice exhibited a distinct shift in gut microbiota, with an increased abundance of beneficial taxa such as *Akkermansia*, *Bifidobacterium*, *Lactococcus*, *Gordonibacter*, and *Turicibacter*, alongside a marked reduction in the pro-inflammatory genus *Escherichia–Shigella.* These microbial alterations have been strongly associated with improvements in metabolic health, gut barrier function, and inflammation mitigation. *Akkermansia*, a mucin-degrading bacterium, is known to enhance intestinal barrier integrity and suppress inflammation [28,48]. *Bifidobacterium* promotes immunomodulation by stimulating interleukin-10-producing dendritic cells [49], while *Lactococcus* and *Gordonibacter* have been implicated in metabolic protection and non-alcoholic fatty liver disease improvement [29,50]. Moreover, *Turicibacter* is reported to influence host immune responses and delay non-alcoholic fatty liver disease progression [51].

We also noted increases in *Candidatus Saccharimonas*, *Eggerthellaceae*, *Parabacteroides*, and *Enterorhabdus*, all of which have been linked to anti-inflammatory activity and metabolic benefits [52,53,54,55]. In contrast, the abundance of *Escherichia–Shigella*, which releases enterotoxins and promotes gut inflammation [27], was significantly reduced in *Antrodia cinnamomea* alcohol extract-treated mice.

Notably, by comparing NCD- and high-fat diet-fed mice, we observed that *Antrodia cinnamomea* alcohol extract-induced microbial alterations were diet-dependent, suggesting that *Antrodia cinnamomea* alcohol extract’s impact on microbiota composition may vary depending on the host’s dietary background—a dimension rarely addressed in prior work. Collectively, these findings indicate that *Antrodia cinnamomea* alcohol extract reconfigures the gut microbiota toward a more metabolically favorable and anti-inflammatory profile, which may contribute to its systemic anti-obesity effects.

Despite these promising findings, several limitations warrant further investigation. First, the causal relationship between microbiota changes and metabolic improvement remains correlative. Fecal microbiota transplantation (FMT) from *Antrodia cinnamomea* alcohol extract-treated mice into germ-free or antibiotic-treated mice could help clarify microbiome-mediated mechanisms. Although our 16S rRNA sequencing results revealed significant taxonomic shifts in the gut microbiota, functional predictions of microbial metabolic capacity, such as through PICRUSt2 analysis, were not performed. The absence of functional metagenomic inference limits our ability to link microbial composition to metabolic outcomes. We acknowledge this as a limitation and suggest that future studies integrate metagenomic or metabolomic approaches to provide a more comprehensive understanding of the gut–metabolism axis.

Second, while our study confirmed AMPK/MAPK modulation during early adipogenesis in vitro, future studies should validate these findings in vivo using conditional knockout mice or pharmacologic inhibitors (e.g., dorsomorphin, U0126). Although our RNA-seq analysis revealed broad transcriptomic changes, qPCR or protein-level validation of key DEGs such as *Adig* and *Scd1* would further strengthen the robustness of our findings. This validation was not conducted in the current study due to sample limitations. Future investigations should include gene-specific confirmation to support these transcriptomic results.

The precise mechanisms underlying the anti-obesity effects of *Antrodia cinnamomea* alcohol extract remain under active investigation. According to our previous study [22], the *Antrodia cinnamomea* alcohol extract preparation used in this study primarily contains triterpenoids and polysaccharides, as identified by LC-MS analysis. These phytochemicals have been reported to exhibit diverse metabolic regulatory functions, including lipid-lowering, anti-inflammatory, and hepatoprotective activities [56,57,58,59,60,61], which may contribute to the observed anti-obesity effects. This implies that the active constituents in *Antrodia cinnamomea* alcohol extract may play a pivotal role in modulating adipogenesis and lipid metabolism. Further investigation into the bioactive compounds of *Antrodia cinnamomea* alcohol extract is warranted. Specifically, triterpenoids such as antcin A, antcin H, antcin K, and zhankuic acid C have demonstrated anti-lipogenic and hepatoprotective properties [62,63,64]. Identification and isolation of these key metabolites may facilitate the development of standardized, mechanism-based formulations for future translational or clinical applications.

In conclusion, our findings indicate that *Antrodia cinnamomea* alcohol extract modulates key molecular and microbial pathways to exert anti-obesity effects. The novelty of this study lies in its integrated multi-omics approach, in vivo tissue-level validation, and the diet-contextual modulation of gut microbiota, collectively providing a comprehensive framework for understanding *Antrodia cinnamomea* alcohol extract as a potential natural therapeutic agent for obesity management.

## 4. Materials and Methods

### 4.1. Preparation of Antrodia cinnamomea Extract (ACE)

Dried mycelia of *Antrodia cinnamomea* (3 kg) were obtained from Lantyng Biotechnology (Taipei, Taiwan). The material was extracted three times with 95% ethanol (30 L per extraction) at room temperature. The combined extracts were filtered and concentrated under reduced pressure to yield a brown syrup (1713.49 g), as previously reported [22].

### 4.2. 3T3-L1 Cell Culture and Adipocyte Differentiation

Mouse 3T3-L1 preadipocytes (ATCC, Manassas, VA, USA) were cultured in Dulbecco’s modified Eagle’s medium (DMEM) supplemented with 10% fetal bovine serum (FBS), 100 U/mL penicillin, and 100 µg/mL streptomycin at 37 °C in 5% CO_2_. To induce adipocyte differentiation, confluent cells were treated with induction medium containing 0.25 mM 3-isobutyl-1-methylxanthine, 1 µM dexamethasone, 1 µg/mL insulin, and 2 µM rosiglitazone (M.D.I.R) for 4 days, with media refreshed every 2 days. Differentiation was continued with insulin-only medium (1 µg/mL) for an additional 2 days [23,65,66].

### 4.3. Animal Experiments

Seven-week-old male C57BL/6 mice (weight: 21–25 g) were randomly divided into four groups: normal chow diet + vehicle (NCD, n = 13), normal chow diet + *Antrodia cinnamomea* alcohol extract (NCD + ACE, n = 13), high-fat diet + vehicle (HFD, n = 14), and high-fat diet + *Antrodia cinnamomea* alcohol extract (HFD + ACE, n = 15). *Antrodia cinnamomea* alcohol extract was administered to mice via drinking water at a dosage of 100 mg/kg/day for a period of 10 weeks. This regimen was designed to mimic a realistic intake scenario for potential therapeutic applications. The starting point of *Antrodia cinnamomea* alcohol extract administration was at the beginning of the high-fat diet feeding regimen, which lasted for the entire 10-week study period. Mice were weighed weekly, and blood, liver, and paratesticular adipose tissues were collected at sacrifice. All procedures were approved by the Institutional Animal Care and Use Committee of the National Defense Medical Center (IACUC-19-089).

### 4.4. Cell Viability Assay

*Antrodia cinnamomea* alcohol extract cytotoxicity was evaluated using a Cell Counting Kit-8 (CCK-8; Dojindo, Tokyo, Japan). Cells (1 × 10^4^/well) were seeded in 96-well plates and treated with varying *Antrodia cinnamomea* alcohol extract concentrations dissolved in DMSO (final DMSO concentration 0.1%) for 24 h. CCK-8 reagent was added and incubated for 2 h. Absorbance was read at 450/650 nm using a microplate reader (SpectraMax 190, Molecular Devices, Sunnyvale, CA, USA).

### 4.5. Oil Red O Staining

Lipid accumulation was assessed by oil red O staining. Differentiated cells were fixed with 10% formalin in PBS at 4 °C for 1 h, washed twice with PBS, and stained with 0.6% oil red O for 15 min at room temperature. For 3T3-L1 cells, intracellular lipids were extracted with isopropanol, and absorbance was measured at 500 nm using a spectrophotometer (Spectra Max 190; Molecular Devices, Sunnyvale, CA, USA). For liver tissue sections, stained lipid droplets were visualized under a light microscope, and representative images were captured for comparison between groups.

### 4.6. Protein Extraction and Western Blotting

Protein extraction and Western blotting were performed as previously described [67]. Briefly, total protein lysates were prepared by incubating cells in 200 µL of Radio-Immunoprecipitation Assay (RIPA) buffer (Sigma-Aldrich, St. Louis, MO, USA) supplemented with protease and phosphatase inhibitor cocktails. Protein concentration was determined using a BCA assay kit (Pierce, Rockford, IL, USA). Equal amounts of protein (20–40 µg) were separated on SDS-PAGE gels and transferred to PVDF membranes (Millipore, Bedford, MA, USA). Membranes were blocked in 5% non-fat milk for 1 h at room temperature and subsequently incubated overnight at 4 °C with primary antibodies targeting SREBP1, C/EBPα, AMPK, phosphorylated AMPK (Thr172), ACC, phosphorylated ACC (Ser79), p-FOXO1 (Ser256), ERK, phosphorylated ERK (Thr202/Tyr204), p38, phosphorylated p38 (Thr180/Tyr182), JNK, phosphorylated JNK (Thr183/Tyr185), PPARγ (all from Cell Signaling Technology, Boston, MA, USA; 1:1000 dilution for all except adiponectin at 1:500), and FASN and GAPDH (from Proteintech, Rosemont, IL, USA; 1:1000 or 1:10,000 dilution, respectively). The following day, membranes were incubated with HRP-conjugated secondary antibodies—anti-mouse or anti-rabbit (Santa Cruz Biotechnology, Heidelberg, Germany; both at 1:10,000 dilution)—for 1 h at room temperature. Protein bands were visualized using an enhanced chemiluminescence (ECL) detection system (Amersham Biosciences, Piscataway, NJ, USA). Images were captured using a luminescent image analyzer (LAS-3000, Fujifilm, Tokyo, Japan). Band intensities were quantified with ImageJ software (version 1.50a) and normalized to GAPDH or total protein controls.

### 4.7. Biochemical and Histological Analyses

Serum levels of aspartate aminotransferase (AST), alanine aminotransferase (ALT), creatinine (CREA), total cholesterol (T-CHO), triglyceride (TG), low-density lipoprotein (LDL), and high-density lipoprotein (HDL) were measured by the National Laboratory Animal Center. For histological examination, one-third of the largest hepatic lobe was embedded in Tissue-Tek O.C.T. compound and rapidly frozen in liquid nitrogen, then stored at −80 °C. Frozen sections were prepared using a cryostat and stained with oil red O to evaluate hepatic lipid accumulation. The remaining liver tissues were fixed in 10% neutral-buffered formalin, embedded in paraffin, and sectioned for hematoxylin and eosin (H&E) staining. non-alcoholic fatty liver disease activity score (NAS) was used to assess liver pathology [26].

### 4.8. Micro-Computed Tomography

Micro-computed tomography scanning was performed with 360° X-ray scanning in the 0.01–10 nm range. The reconstructed 3D images were analyzed to evaluate visceral fat accumulation based on density contrast between tissues. Variations in tissue density are depicted in shades ranging from white to black. Soft tissues, such as fat, appear white-gray in the images owing to their specific density characteristics.

### 4.9. RNA Sequencing and Transcriptome Analysis

Total RNA was extracted from paratesticular adipose tissue and submitted to Toolsbiotech Biotechnology Co., Ltd. (Taipei, Taiwan) for transcriptome profiling. RNA sequencing data quality was first assessed using FastQC v0.12.1 followed by trimming of low-quality bases and adapters using Trimmomatic (v0.38) to generate clean reads. The clean reads were then aligned to the reference mouse genome using HISAT2 (v2.1.0), and gene-level counts were obtained using featureCounts (v2.0.0). Differentially expressed genes (DEGs) were identified using the DESeq2 package in v1.26.0, employing relative log expression (RLE) normalization. To control for false discovery, *p*-values were adjusted using the Benjamini–Hochberg method, and genes with adjusted *p*-values (FDR) < 0.05 and |log_2_ fold change| > 1 were considered significantly differentially expressed.

Functional enrichment analysis of DEGs was performed using clusterProfiler (v3.14.3), with annotation to Gene Ontology (GO) and Kyoto Encyclopedia of Genes and Genomes (KEGG) databases. These analyses were conducted to identify key biological processes and pathways modulated by *Antrodia cinnamomea* alcohol extract treatment, particularly those involved in adipogenesis and lipid metabolism. The fold change represents the magnitude of expression difference between treatment groups, while the *p*-value reflects statistical significance, with smaller *p*-values indicating greater confidence in differential expression.

### 4.10. Gut Microbiota Analysis

Fecal samples were collected from individual mice at the end of the experiment for microbial community analysis. DNA was extracted and amplified using primers targeting the V3–V4 hypervariable regions of the bacterial 16S rRNA gene. Amplicon libraries were constructed and sequenced using the Illumina MiSeq platform (Illumina, San Diego, CA, USA) with paired-end reads. The raw sequencing data were quality-filtered, merged, and clustered into operational taxonomic units (OTUs) based on sequence similarity. Taxonomic assignment was performed by comparing representative sequences to the SILVA reference database. Microbial diversity was evaluated by calculating alpha diversity indices (e.g., Shannon index) to assess species richness within samples, and beta diversity was assessed to evaluate differences in community composition between groups. Principal component analysis (PCA) was used to visualize clustering patterns of microbiota structure, and linear discriminant analysis effect size (LEfSe) was employed to identify significantly different taxa associated with *Antrodia cinnamomea* alcohol extract treatment or dietary conditions.

### 4.11. Statistical Analysis

All data are represented as the mean ± standard error of the mean (SEM). Statistical differences among groups were evaluated using one-way analysis of variance (ANOVA), followed by Bonferroni post hoc tests. All analyses were conducted using IBM SPSS Statistics version 22 (IBM^®^ SPSS^®^ Statistics 22, Armonk, NY, USA). A *p*-value of <0.05 was considered statistically significant. Normality testing was not performed, which is acknowledged as a limitation in the statistical interpretation of the results. 

## 5. Conclusions

In conclusion, this study provides comprehensive evidence that *Antrodia cinnamomea* extract (ACE) exerts multi-level anti-obesity effects by regulating lipid metabolism, suppressing adipogenesis, and remodeling gut microbiota. Through both in vitro and in vivo approaches, we demonstrated that *Antrodia cinnamomea* alcohol extract modulates the AMPK and MAPK signaling pathways, downregulates key lipogenic and adipogenic genes, and improves metabolic parameters in high-fat diet-fed mice. Importantly, our study is among the first to integrate protein-level and transcriptome-level analyses of adipose tissue with gut microbiota profiling, thereby offering a comprehensive view of *Antrodia cinnamomea* alcohol extract’s physiological effects.

## Data Availability

The datasets used and/or analyzed during the current study are available from the corresponding author upon reasonable request.

## Supplementary Materials

The following supporting information can be downloaded at: https://www.mdpi.com/article/10.3390/ijms26125856/s1.
