# Supplementary material for: Antrodia cinnamomea Extract Attenuates Obesity by Targeting Adipogenic Pathways and Gut Dysbiosis in High-Fat Diet-Fed Mice
Source: Int J Mol Sci. 2025 Jun 18;26(12):5856. doi: 10.3390/ijms26125856 (PMC12193264; doi:10.3390/ijms26125856)
Supplement: Supplementary file 1 [file ijms-26-05856-s001.zip › ijms-3619130-supplementary.pdf]

Supplementary Figures

Figure S1

A

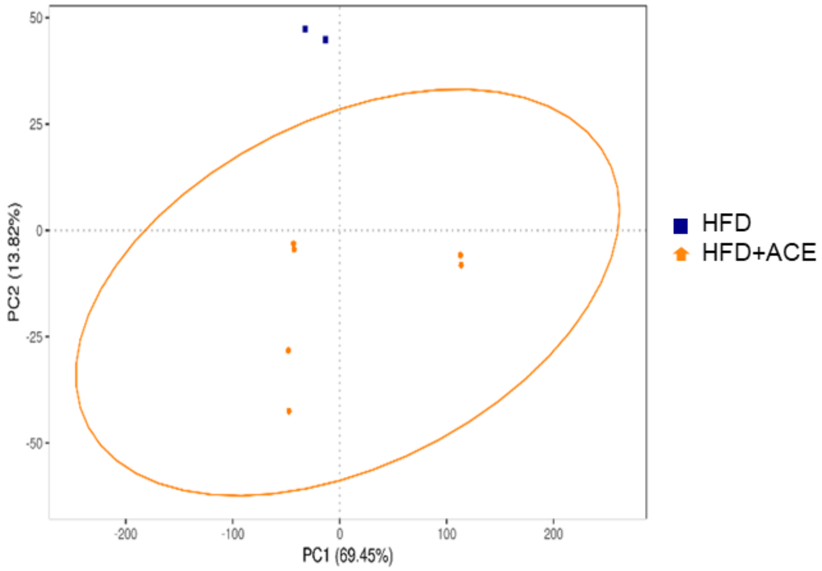

B

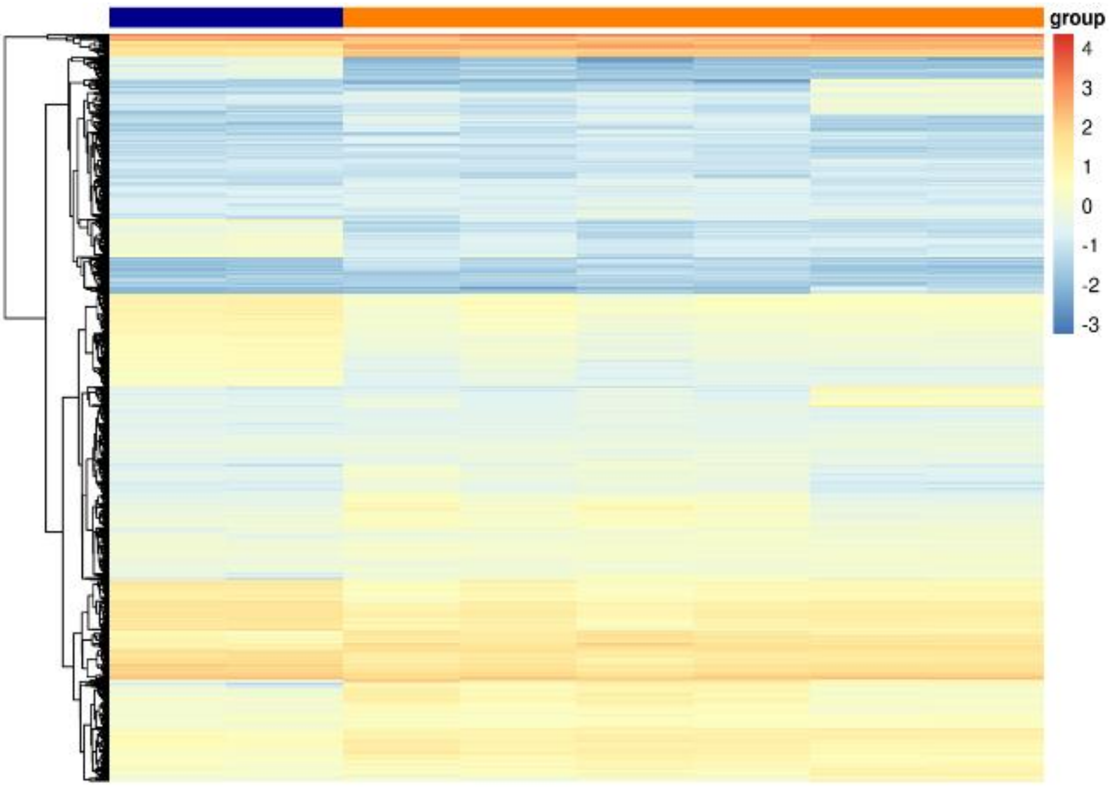

Figure S2

A

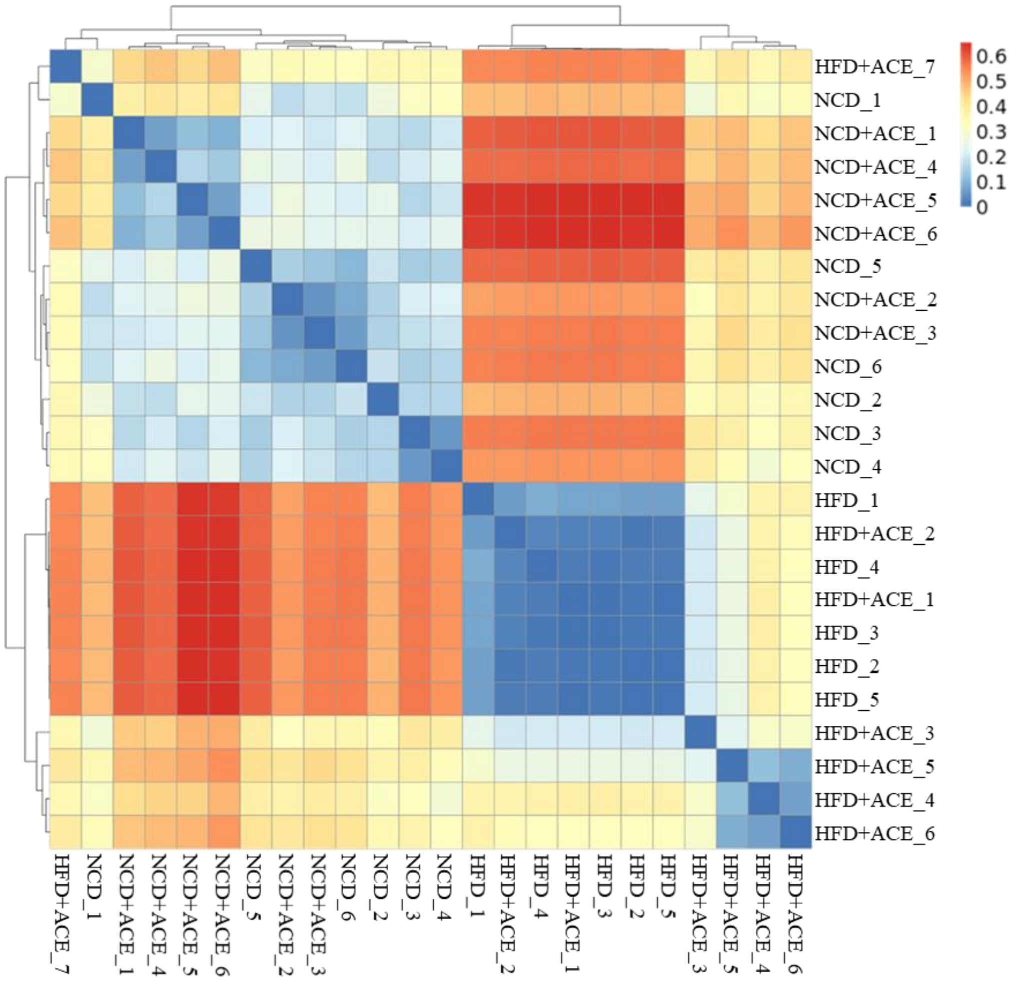
HFD+ACE_7
NCD_1
NCD+ACE_1
NCD+ACE_4
NCD+ACE_5
NCD+ACE_6
NCD_5
NCD+ACE_2
NCD+ACE_3
NCD_6
NCD_2
NCD_3
NCD_4
HFD_1
HFD+ACE_2
HFD_4
HFD+ACE_1
HFD_3
HFD_2
HFD_5
HFD+ACE_3
HFD+ACE_5
HFD+ACE_4
HFD+ACE_6
0.6
0.5
0.4
0.3
0.2
0.1
0

B

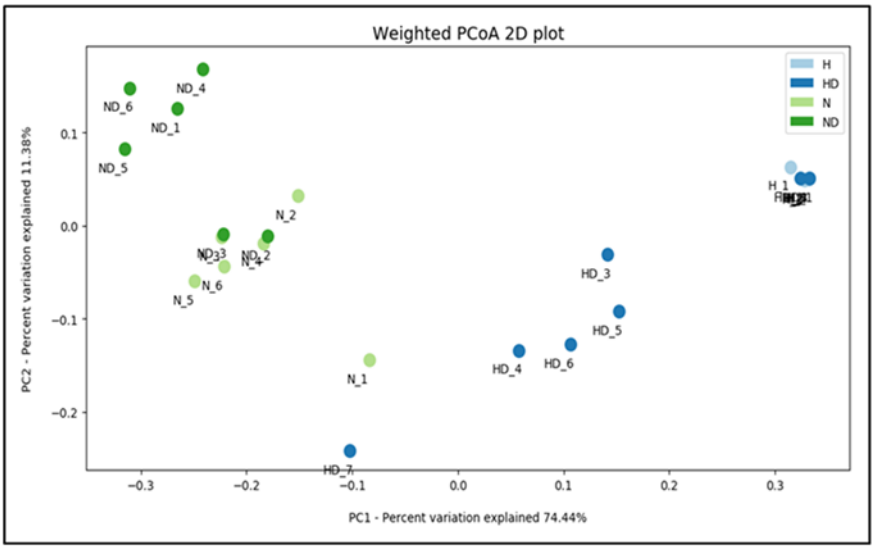
Weighted PCoA 2D plot
H
HD
N
ND
PC2 - Percent variation explained 11.38%
PC1 - Percent variation explained 74.44%
ND_4
ND_6
ND_1
ND_5
N_2
N_5
N_6
N_1
HD_3
HD_5
HD_6
HD_4
HD_7
H_1

C

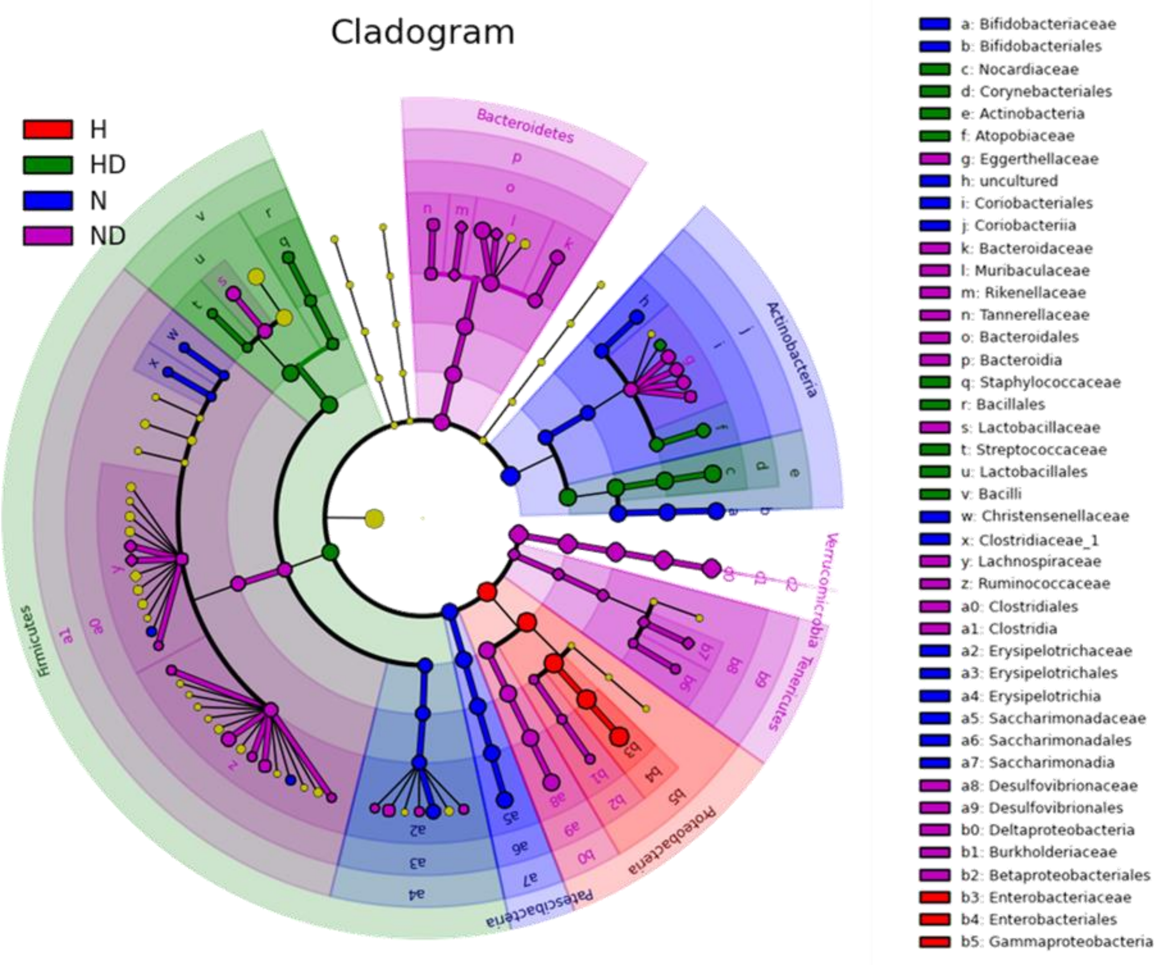
Cladogram
H
HD
N
ND
Bacteroidetes
Actinobacteria
Verrucomicrobia
Tenericutes
Proteobacteria
Patescibacteria
Firmicutes
a: Bifidobacteriaceae
b: Bifidobacteriales
c: Nocardiaceae
d: Corynebacteriales
e: Actinobacteria
f: Atopobiaceae
g: Eggerthellaceae
h: uncultured
i: Coriobacteriales
j: Coriobacteriia
k: Bacteroidaceae
l: Muribaculaceae
m: Rikenellaceae
n: Tannerellaceae
o: Bacteroidales
p: Bacteroidia
q: Staphylococcaceae
r: Bacillales
s: Lactobacillaceae
t: Streptococcaceae
u: Lactobacillales
v: Bacilli
w: Christensenellaceae
x: Clostridiaceae_1
y: Lachnospiraceae
z: Ruminococcaceae
a0: Clostridiales
a1: Clostridia
a2: Erysipelotrichaceae
a3: Erysipelotrichales
a4: Erysipelotrichia
a5: Saccharimonadaceae
a6: Saccharimonadales
a7: Saccharimonadia
a8: Desulfovibrionaceae
a9: Desulfovibrionales
b0: Deltaproteobacteria
b1: Burkholderiaceae
b2: Betaproteobacteriales
b3: Enterobacteriaceae
b4: Enterobacteriales
b5: Gammaproteobacteria

Figure S3

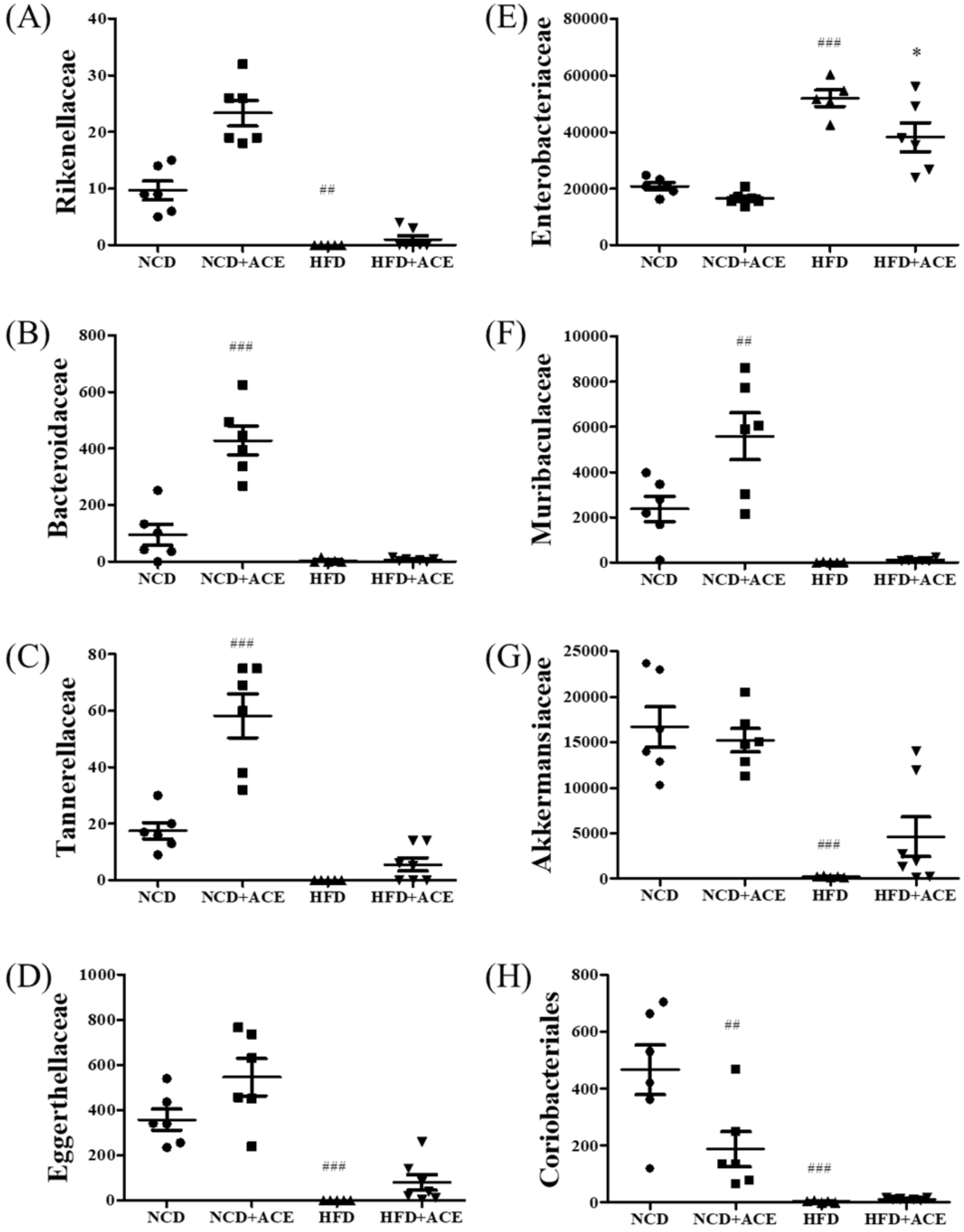

(A)
Rikenellaceae
40
30
20
10
0
NCD
NCD+ACE
HFD
HFD+ACE
##
(B)
Bacteroidaceae
800
600
400
200
0
NCD
NCD+ACE
HFD
HFD+ACE
###
(C)
Tannerellaceae
80
60
40
20
0
NCD
NCD+ACE
HFD
HFD+ACE
###
(D)
Eggerthellaceae
1000
800
600
400
200
0
NCD
NCD+ACE
HFD
HFD+ACE
###
(E)
Enterobacteriaceae
80000
60000
40000
20000
0
NCD
NCD+ACE
HFD
HFD+ACE
###
*
(F)
Muribaculaceae
10000
8000
6000
4000
2000
0
NCD
NCD+ACE
HFD
HFD+ACE
##
(G)
Akkermansiaceae
25000
20000
15000
10000
5000
0
NCD
NCD+ACE
HFD
HFD+ACE
###
(H)
Coriobacteriales
800
600
400
200
0
NCD
NCD+ACE
HFD
HFD+ACE
##
###

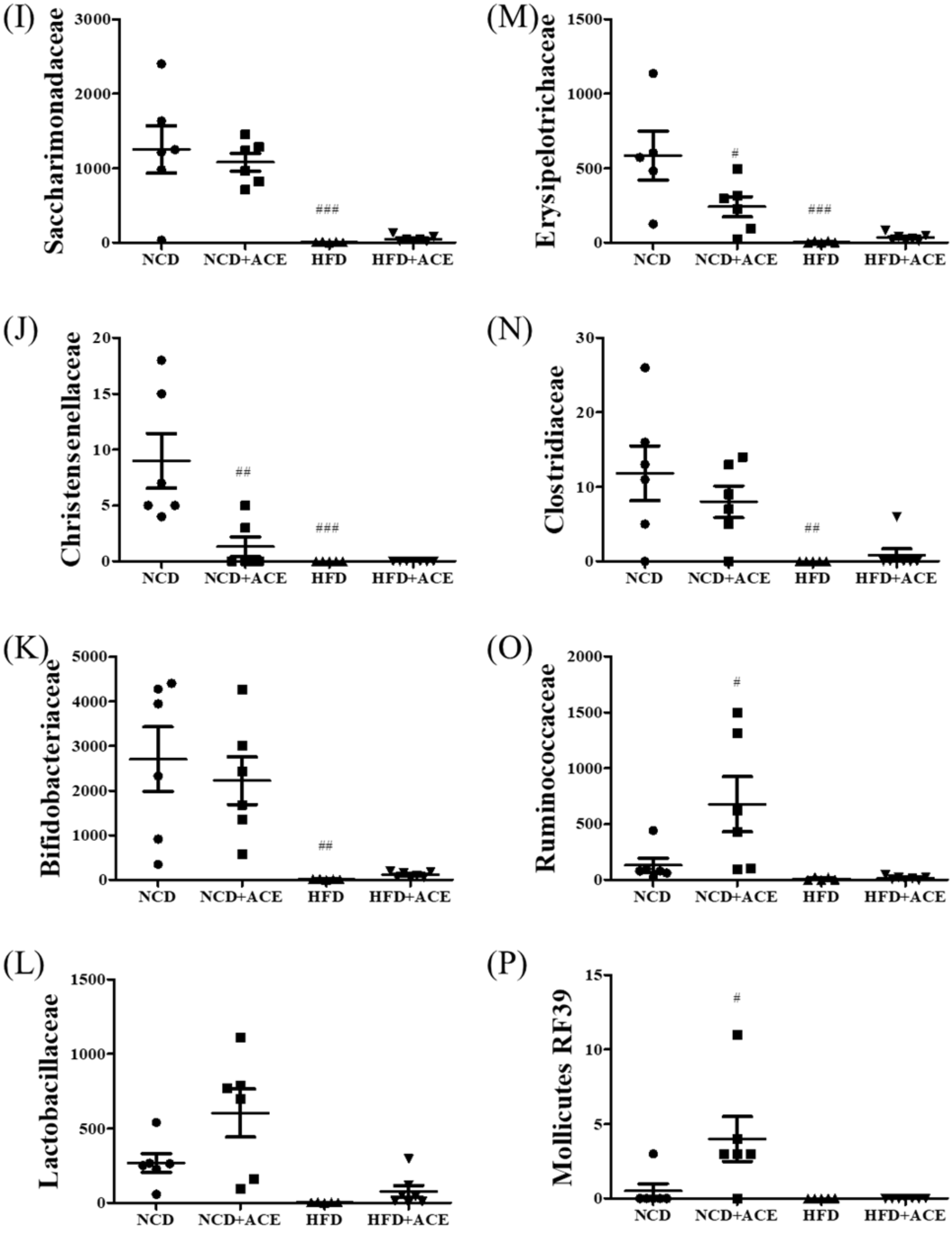
(I)
Saccharimonadaceae
3000
2000
1000
0
###
NCD
NCD+ACE
HFD
HFD+ACE
(M)
Erysipelotrichaceae
1500
1000
500
0
#
###
NCD
NCD+ACE
HFD
HFD+ACE
(J)
Christensenellaceae
20
15
10
5
0
##
###
NCD
NCD+ACE
HFD
HFD+ACE
(N)
Clostridiaceae
30
20
10
0
##
NCD
NCD+ACE
HFD
HFD+ACE
(K)
Bifidobacteriaceae
5000
4000
3000
2000
1000
0
##
NCD
NCD+ACE
HFD
HFD+ACE
(O)
Ruminococcaceae
2000
1500
1000
500
0
#
NCD
NCD+ACE
HFD
HFD+ACE
(L)
Lactobacillaceae
1500
1000
500
0
NCD
NCD+ACE
HFD
HFD+ACE
(P)
Mollicutes RF39
15
10
5
0
#
NCD
NCD+ACE
HFD
HFD+ACE

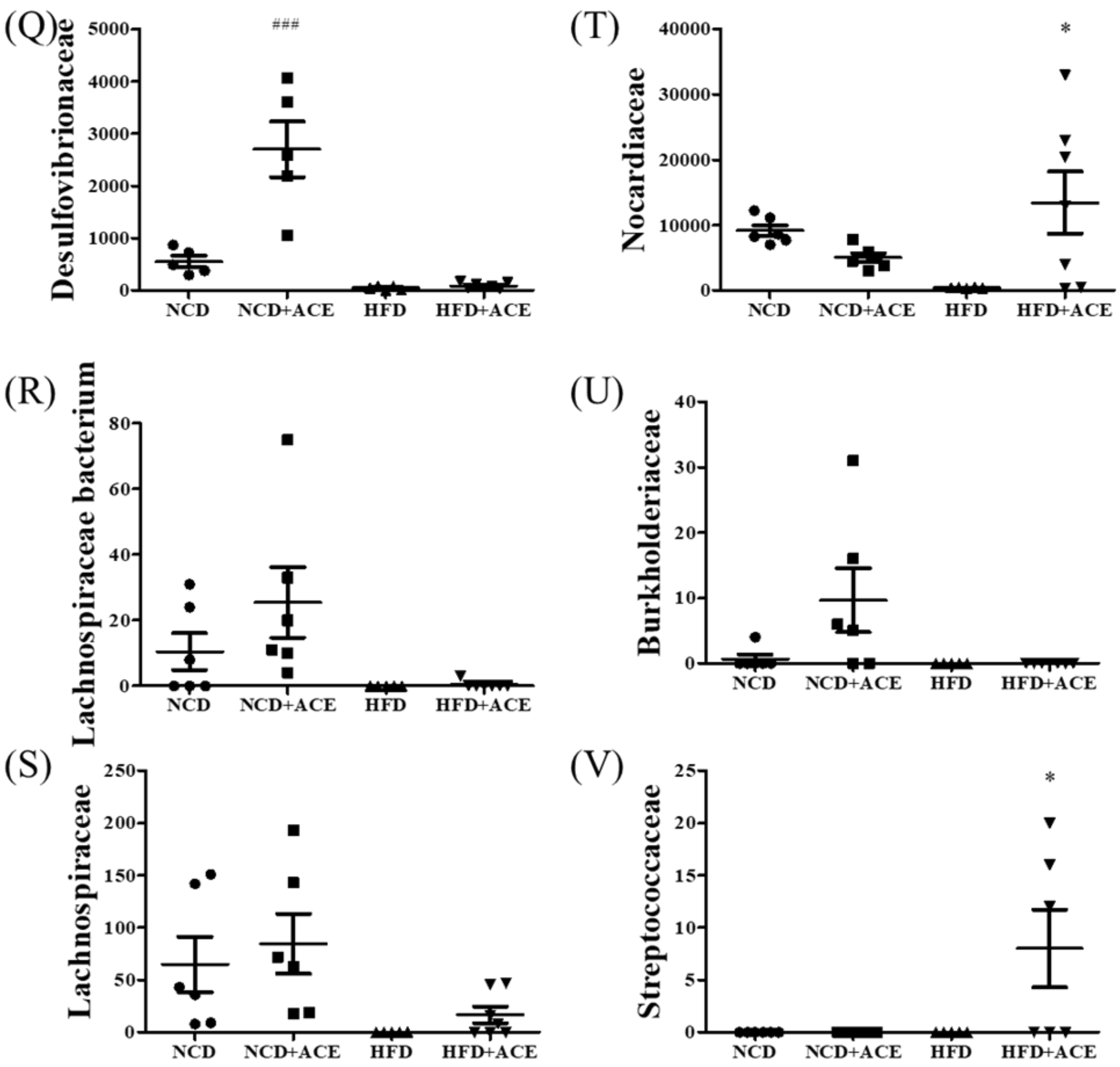
(Q)
Desulfovibrionaceae
###
0
1000
2000
3000
4000
5000
NCD
NCD+ACE
HFD
HFD+ACE
(T)
Nocardiaceae
*
0
10000
20000
30000
40000
NCD
NCD+ACE
HFD
HFD+ACE
(R)
Lachnospiraceae bacterium
0
20
40
60
80
NCD
NCD+ACE
HFD
HFD+ACE
(U)
Burkholderiaceae
0
10
20
30
40
NCD
NCD+ACE
HFD
HFD+ACE
(S)
Lachnospiraceae
0
50
100
150
200
250
NCD
NCD+ACE
HFD
HFD+ACE
(V)
Streptococcaceae
*
0
5
10
15
20
25
NCD
NCD+ACE
HFD
HFD+ACE

Figure S4

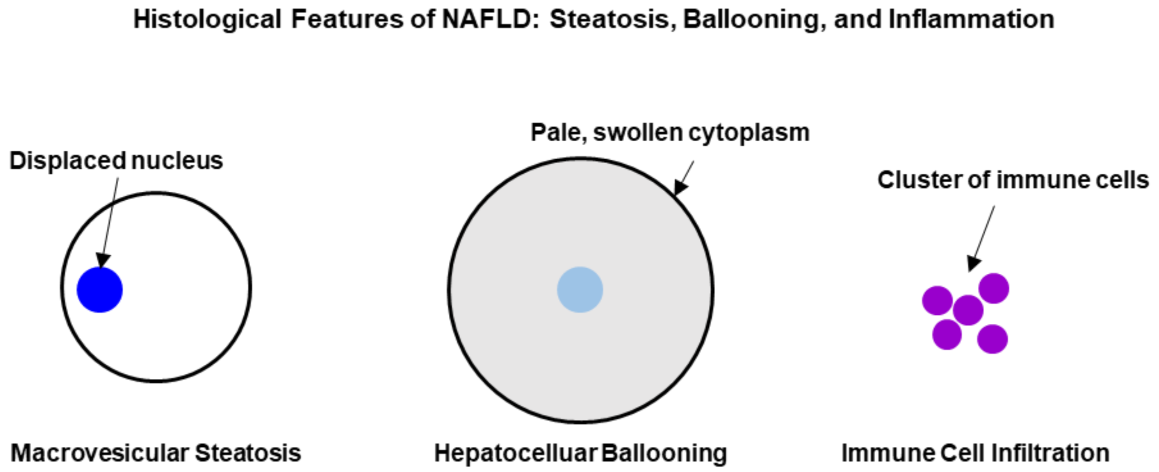
Histological Features of NAFLD: Steatosis, Ballooning, and Inflammation
Displaced nucleus
Pale, swollen cytoplasm
Cluster of immune cells
Macrovesicular Steatosis
Hepatocelluar Ballooning
Immune Cell Infiltration

Supplementary figure legends

Figure S1. Transcriptomic overview of adipose tissue gene expression in HFD and HFD+ACE groups. (A) Principal Component Analysis (PCA) showing distinct clustering of HFD and HFD+ACE samples based on global transcriptomic profiles. **(B)** Heatmap of hierarchical clustering for the top 50 differentially expressed genes based on normalized transcript counts. Each column represents an individual mouse.

Figure S2. Gut microbiota structure and discriminative taxa following ACE treatment. (A) Pairwise Bray–Curtis dissimilarity heatmap comparing gut microbiota composition among all individual samples. Darker colors indicate higher similarity (lower dissimilarity), lighter colors indicate greater dissimilarity. **(B)** PCA plot based on microbial community composition showing group-specific clustering (NCD, NCD+ACE, HFD, HFD+ACE). Each dot represents one mouse. **(C)** Cladogram generated by LEfSe analysis showing the phylogenetic distribution of taxa differentially abundant between HFD and HFD+ACE groups.

Figure S3. Relative abundance of major gut microbiota taxa at the genus level. Abundance levels of 22 bacterial taxa at the family or order level were compared across the four experimental groups (NCD, NCD+ACE, HFD, and HFD+ACE). (A) *Rikenellaceae*, (B) *Bacteroidaceae*, (C) *Tannerellaceae*, (D) *Eggerthellaceae*, (E) *Enterobacteriaceae*, (F) *Muribaculaceae*, (G) *Akkermansiaceae*, (H) *Coriobacteriales*,

(I) *Saccharimonadaceae*, (J) *Christensenellaceae*, (K) *Bifidobacteriaceae*, (L) *Lactobacillaceae*, (M) *Erysipelotrichaceae*, (N) *Clostridiaceae*, (O) *Ruminococcaceae*, (P) *Mollicutes RF39*, (Q) *Desulfovibrionaceae*, (R) *Lachnospiraceae bacterium*, (S) *Lachnospiraceae*, (T) *Nocardiaceae*, (U) *Burkholderiaceae*, (V) *Streptococcaceae*. Each dot represents an individual mouse; error bars represent the mean ± SEM. Significant differences between groups were determined by appropriate statistical tests. #$p < 0.05$, ##$p < 0.01$, ###$p < 0.001$ vs. NCD; *$p < 0.05$, **$p < 0.01$, ***$p < 0.001$ vs. HFD.

Figure S4. Histological illustration of key NAFLD features: macrovesicular steatosis, hepatocellular ballooning, and immune cell infiltration. Schematic representations of the three principal hepatic histopathological features used in NAFLD assessment. **Left**: Macrovesicular steatosi*s* is characterized by a large intracellular lipid droplet displacing the hepatocyte nucleus toward the periphery. **Center**: Hepatocellular ballooning presents as swollen hepatocytes with pale, rarefied cytoplasm and a centrally located nucleus. **Right**: Immune cell infiltration is represented by clusters of small, round, dark-staining lymphocyte-like cells accumulating in the hepatic parenchyma.

Supplementary Tables

Table S1. Gene expression of fatty acid metabolic process on GO analysis.

fatty acid metabolic process				
No.	Gene name	Gene description	log2 FC	P.adj
1	Por	P450 (cytochrome) oxidoreductase [Source:MGI Symbol;Acc:MGI:97744]	-1.12	0.0006
2	Acaca	acetyl-Coenzyme A carboxylase alpha [Source:MGI Symbol;Acc:MGI:108451]	-1.27	0.0013
3	Eci3	enoyl-Coenzyme A delta isomerase 3 [Source:MGI Symbol;Acc:MGI:1916373]	-2.38	0.0002
4	Gpam	glycerol-3-phosphate acyltransferase, mitochondrial [Source:MGI Symbol;Acc:MGI:109162]	-1.40	0.001
5	Cyp2e1	cytochrome P450, family 2, subfamily e, polypeptide 1 [Source:MGI Symbol;Acc:MGI:88607]	-3.08	0.0098
6	Phyh	phytanoyl-CoA hydroxylase [Source:MGI Symbol;Acc:MGI:891978]	-1.34	3E-11
7	Ivd	isovaleryl coenzyme A dehydrogenase [Source:MGI Symbol;Acc:MGI:1929242]	-1.28	2E-05
8	Pck1	phosphoenolpyruvate carboxykinase 1, cytosolic [Source:MGI Symbol;Acc:MGI:97501]	-2.26	2E-18
9	Nr4a3	nuclear receptor subfamily 4, group A, member 3 [Source:MGI Symbol;Acc:MGI:1352457]	-2.45	4E-08
10	Echdc2	enoyl Coenzyme A hydratase domain containing 2 [Source:MGI Symbol;Acc:MGI:1289238]	-1.48	0.0005
11	Acadsb	acyl-Coenzyme A dehydrogenase, short/branched chain [Source:MGI Symbol;Acc:MGI:1914135]	-1.34	2E-05
12	Acsm3	acyl-CoA synthetase medium-chain family member 3 [Source:MGI Symbol;Acc:MGI:99538]	-4.28	5E-07
13	Acsm5	acyl-CoA synthetase medium-chain family member 5 [Source:MGI Symbol;Acc:MGI:2444086]	-1.58	0.0047
14	Ces1f	carboxylesterase 1F [Source:MGI Symbol;Acc:MGI:2142687]	-1.62	0.001
15	Abhd5	abhydrolase domain containing 5 [Source:MGI Symbol;Acc:MGI:1914719]	-1.38	8E-11

Table S2. Gene expression of fat cell differentiation on GO analysis.

fat cell differentiation				
No.	Gene name	Gene description	log2 FC	P.adj
1	Sh2b2	SH2B adaptor protein 2 [Source:MGI Symbol;Acc:MGI:1345171]	-1.24	0.00422
2	Retn	resistin [Source:MGI Symbol;Acc:MGI:1888506]	-1.22	0.04635
3	Xbp1	X-box binding protein 1 [Source:MGI Symbol;Acc:MGI:98970]	-1.20	0.012
4	Sav1	salvador family WW domain containing 1 [Source:MGI Symbol;Acc:MGI:1927144]	-1.34	0.02996
5	Aldh6a1	aldehyde dehydrogenase family 6, subfamily A1 [Source:MGI Symbol;Acc:MGI:1915077]	-1.58	3.9E-08
6	Ebf2	early B cell factor 2 [Source:MGI Symbol;Acc:MGI:894332]	-1.28	0.0007
7	Osbpl11	oxysterol binding protein-like 11 [Source:MGI Symbol;Acc:MGI:2146553]	-1.38	1.1E-09
8	Lgals12	lectin, galactose binding, soluble 12 [Source:MGI Symbol;Acc:MGI:1929094]	-1.56	0.00015
9	Tcf7l2	transcription factor 7 like 2, T cell specific, HMG box [Source:MGI Symbol;Acc:MGI:1202879]	-1.06	0.00075
10	Rorc	RAR-related orphan receptor gamma [Source:MGI Symbol;Acc:MGI:104856]	-3.06	1.8E-06
11	Nr4a3	nuclear receptor subfamily 4, group A, member 3 [Source:MGI Symbol;Acc:MGI:1352457]	-2.45	4.2E-08
12	Adrb3	adrenergic receptor, beta 3 [Source:MGI Symbol;Acc:MGI:87939]	-2.80	0.0043
13	Scd1	stearoyl-Coenzyme A desaturase 1 [Source:MGI Symbol;Acc:MGI:98239]	-1.44	0.01488
14	Adig	adipogenin [Source:MGI Symbol;Acc:MGI:2675492]	-1.03	0.00022
15	Zfp36	zinc finger protein 36 [Source:MGI Symbol;Acc:MGI:99180]	-1.31	0.03719
16	Adrb2	adrenergic receptor, beta 2 [Source:MGI Symbol;Acc:MGI:87938]	-2.96	6.2E-08
17	Ffar4	free fatty acid receptor 4 [Source:MGI Symbol;Acc:MGI:2147577]	-1.32	0.00087
18	Per2	period circadian clock 2 [Source:MGI Symbol;Acc:MGI:1195265]	-1.24	0.00754
19	Selenbp1	selenium binding protein 1 [Source:MGI Symbol;Acc:MGI:96825]	-1.07	0.01454
20	Cebpd	CCAAT/enhancer binding protein (C/EBP), delta [Source:MGI Symbol;Acc:MGI:103573]	-2.63	2.1E-17
21	Bnip3	BCL2/adenovirus E1B interacting protein 3 [Source:MGI Symbol;Acc:MGI:109326]	-1.27	0.00086

Table S3. Top 10 DEGs which were upregulated in HFD supplemented with ACE compared to HFD mice.

	Gene name	Gene description	log2 FC	*P*.adj
1	Cck	cholecystokinin	8.683936744	0.000682732
2	Myh15	myosin, heavy chain 15	8.229474191	6.16178E-05
3	Gm5150	predicted gene 5150	7.843238553	0.000331907
4	Spata25	spermatogenesis associated 25	7.383055769	0.01740438
5	9130204L05Rik	RIKEN cDNA 9130204L05 gene	7.315585526	0.00026019
6	Oit3	oncoprotein induced transcript 3	7.257872212	0.000291139
7	Car6	carbonic anhydrase 6	7.038764515	0.000686384
8	Gm47507	predicted gene, 47507	6.957268255	0.001898667
9	Arfgef3	ARFGEF family member 3	6.79056654	0.006095711
10	Gpr50	G-protein-coupled receptor 50	6.707775415	4.47306E-14

Table S4. Top 10 DEGs which were downregulated in HFD supplemented with ACE compared to HFD mice.

	Gene name	Gene description	log2 FC	*P*.adj
1	Gm38244	predicted gene, 38244	-5.069627509	0.000735862
2	BB365896	expressed sequence BB365896	-4.946465678	1.20472E-11
3	Grem2	gremlin 2, DAN family BMP antagonist	-4.625192173	2.8318E-17
4	Fam13a	family with sequence similarity 13, member A	-4.361267861	9.16845E-18
5	Irf4	interferon regulatory factor 4	-4.357786604	1.17253E-08
6	Acsm3	acyl-CoA synthetase medium-chain family member 3	-4.284228214	4.97387E-07
7	2010016I18Rik	RIKEN cDNA 2010016I18 gene	-4.22320849	8.64579E-07
8	Ripk4	receptor-interacting serine-threonine kinase 4	-3.627614549	0.000300245
9	Eif4ebp3	eukaryotic translation initiation factor 4E binding protein 3	-3.361620696	0.032726191
10	Dennd2d	DENN/MADD domain containing 2D	-3.237991098	8.16962E-08

Table S5. ACE modulated gut microbiota in HFD-fed C57BL/6 model.

Variable	Group:H	Group:HD	p-value
Escherichia-Shigella	98.56±0.19	53.78±0.96	<0.001
Bifidobacterium	0.03±0.02	0.23±0.10	0.003
Gordonibacter	0.00±0.00	0.01±0.00	0.005
Rhodococcus	0.77±0.11	29.44±7.03	0.006
Muribaculaceae	0.02±0.02	0.16±0.09	0.01
Candidatus Saccharimonas	0.02±0.01	0.10±0.06	0.015
Eggerthellaceae	0.00±0.00	0.02±0.01	0.016
Enterorhabdus	0.00±0.00	0.06±0.04	0.02
Parabacteroides	0.00±0.00	0.01±0.01	0.03
Turicibacter	0.01±0.01	0.03±0.01	0.035
Akkermansia	0.43±0.15	9.47±0.37	0.042
Coriobacteriales	0.01±0.01	0.02±0.01	0.042
Lactococcus	0.00±0.00	0.02±0.01	0.045

H: high-fat-diet group; HD: high-fat-diet with ACE treatment

Table S6. ACE modulates gut microbiota in NCD-fed C57BL/6 model.

Variable	Group:N	Group:ND	p-value
Bacteroides	0.16±0.14	0.83±0.22	0.002
Enterorhabdus	0.11±0.04	0.24±0.07	0.002
Parabacteroides	0.03±0.01	0.11±0.03	0.002
Alistipes	0.02±0.01	0.05±0.01	0.002
Rhodococcus	16.13±3.75	9.52±2.45	0.009
Turicibacter	1.21±0.99	0.20±0.23	0.009
uncultured Bacteroidales bacterium	0.00±0.00	0.03±0.02	0.013
Mollicutes RF39	0.00±0.00	0.01±0.01	0.013
Christensenellaceae	0.02±0.01	0.00±0.00	0.014
Eggerthellaceae	0.10±0.06	0.29±0.17	0.015
Ruminococcus gauvreauii group	0.01±0.01	0.00±0.00	0.022
Faecalibaculum	0.00±0.00	0.01±0.01	0.022
Lachnospiraceae	0.00±0.00	0.01±0.01	0.022
Ruminococcaceae UCG-013	0.01±0.01	0.02±0.01	0.025
Muribaculaceae	4.17±2.44	10.59±4.49	0.026
Desulfovibrio	3.08±5.18	6.74±4.18	0.041
Ruminiclostridium 5	0.15±0.28	1.18±1.18	0.041
Erysipelotrichaceae	0.00±0.01	0.02±0.01	0.049
Parasutterella	0.00±0.00	0.02±0.02	0.05

N: normal chow diet group; NCD: normal chow diet with ACE treatment
